# Identification, Molecular Cloning, and Functional Characterization of a Coniferyl Alcohol Acyltransferase Involved in the Biosynthesis of Dibenzocyclooctadiene Lignans in *Schisandra chinensis*

**DOI:** 10.3389/fpls.2022.881342

**Published:** 2022-06-23

**Authors:** Ting-Yan Qiang, Jiu-Shi Liu, Yu-Qing Dong, Xin-Lu Mu, Yu Chen, Hong-Mei Luo, Ben-Gang Zhang, Hai-Tao Liu

**Affiliations:** ^1^Bioactive Substances and Resources Utilization of Chinese Herbal Medicine, Ministry of Education, Institute of Medicinal Plant Development, Chinese Academy of Medical Sciences & Peking Union Medical College, Beijing, China; ^2^Engineering Research Center of Tradition Chinese Medicine Resource, Ministry of Education, Institute of Medicinal Plant Development, Chinese Academy of Medical Sciences & Peking Union Medical College, Beijing, China

**Keywords:** *Schisandra chinensis*, coniferyl alcohol, coniferyl alcohol acyltransferase, BAHD acyltransferase family, dibenzocyclooctadiene lignans

## Abstract

*Schisandra chinensis* owes its therapeutic efficacy to the dibenzocyclooctadiene lignans, which are limited to the Schisandraceae family and whose biosynthetic pathway has not been elucidated. Coniferyl alcohol is the synthetic precursor of various types of lignans and can be acetylated to form coniferyl acetate by coniferyl alcohol acyltransferase (CFAT), which belongs to the BAHD acyltransferase family. This catalytic reaction is important because it is the first committed step of the hypothetical biosynthetic pathway in which coniferyl alcohol gives rise to dibenzocyclooctadiene lignans. However, the gene encoding CFAT in *S. chinensis* has not been identified. In this study, firstly we identified 37 *ScBAHD* genes from the transcriptome datasets of *S. chinensis*. According to bioinformatics, phylogenetic, and expression profile analyses, 1 BAHD gene, named *ScBAHD1*, was cloned from *S. chinensis*. The heterologous expression in *Escherichia coli* and *in vitro* activity assays revealed that the recombinant enzyme of ScBAHD1 exhibits acetyltransferase activity with coniferyl alcohol and some other alcohol substrates by using acetyl-CoA as the acetyl donor, which indicates ScBAHD1 functions as ScCFAT. Subcellular localization analysis showed that ScCFAT is mainly located in the cytoplasm. In addition, we generated a three-dimensional (3D) structure of ScCFAT by homology modeling and explored the conformational interaction between protein and ligands by molecular docking simulations. Overall, this study identified the first enzyme with catalytic activity from the Schisandraceae family and laid foundations for future investigations to complete the biosynthetic pathway of dibenzocyclooctadiene lignans.

## Introduction

*Schisandra chinensis* (Turcz.) Baill. (Schisandraceae), a deciduous woody vine plant is mainly distributed in north-eastern China, Korea, Japan, and the eastern part of Russia (Hancke et al., 1999). The fruits of *S. chinensis* have been used as traditional Chinese medicine and functional food for 1,000 of years for the effects of astringent, tonifying qi, replenishing production of body fluid, and nourishing kidney to calm heart (Zhai et al., 2018). In addition, the fruits of *S. chinensis* have long been documented in the pharmacopeias of Russia (1990), America (1999), Korea (2002), Japan (2006), WHO (2007), and Europe (2008) (Szopa et al., 2017).

Extensive studies have indicated that the major bioactive components of *S. chinensis* are dibenzocyclooctadiene lignans (aka schisandra lignans; Szopa et al., 2017), which exhibit a wide variety of biological activities, such as anti-liver injury, anti-oxidant, anti-asthmatic, anti-inflammatory, sedation on the central nervous system activities, and so on (Opletal et al., 2004). The hepatoprotective effect is considered to be the principal one, and the structure-activity relationship suggests that the hepatoprotective activity depends on the configuration of biphenyls and the methylenedioxy substituent on the phenyl nucleus (Hikino et al., 1984). In China, two novel anti-hepatitis drugs (bifendate and bicyclol) were sequentially created through synthesizing the analogs of schizandrin C, which is the most effective component against liver injury of *S. chinensis* (Zhu et al., 2019). Apart from the significant pharmacological activities, the phylogenetic distribution of lignan-producing plant species displays that dibenzocyclooctadiene lignans are only found in the Schisandraceae family (Umezawa, 2003).

**Figure 1:**
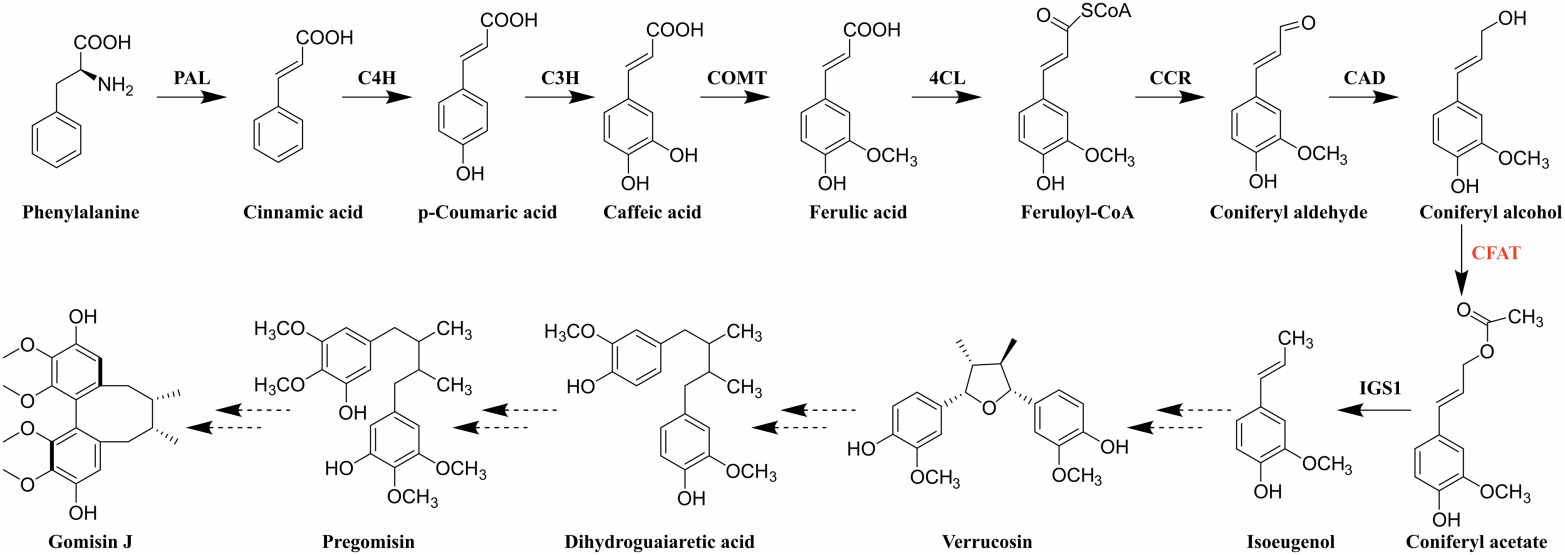
Biosynthetic pathway of dibenzocyclooctadiene lignans. PAL: phenylalanine ammonia-lyase; C4H: cinnamate-4-hydroxylase; C3H: coumarate-3-hydroxylase; COMT: caffeic acid O-methyltransferase; 4CL: 4-hydroxycinnamate CoA ligase; CCR: cinnamoyl-CoA reductase; CAD: cinnamyl alcohol dehydrogenase; CFAT: coniferyl alcohol acyltransferase; and IGS1: isoeugenol synthase 1.

Accordingly, the identification of genes and elucidation of the biosynthetic pathway of dibenzocyclooctadiene lignans have received widespread interest due to the uniqueness of structure, the significance of effect, and the limitation of distribution. Since Erdtman first suggested that the lignan structure was formed by the coupling of two phenylpropanoid monomer units (Erdtman, 1933), the biosynthetic pathway of several typical lignans has been established, such as furofuran, furan, dibenzylbutane, dibenzylbutyrolactone, and aryltetralin lignans (Ono et al., 2006; Lau and Sattely, 2015). In addition, coniferyl alcohol has been proved to be a synthetic precursor of lignans (Suzuki and Umezawa, 2007; Kim et al., 2009). Dexter et al. discovered that coniferyl alcohol could be acetylated to coniferyl acetate by a novel petunia BAHD acyltransferase (PhCFAT; Dexter et al., 2007). And isoeugenol synthase 1 (PhIGS1) was shown to use coniferyl acetate and NADPH as substrates to form *E*-isoeugenol (Koeduka et al., 2006). Previously, Lopes et al. had revealed that *E*-isoeugenol was converted to a furan lignan verrucosin in *Virola surinamensis* through stable-isotope labeling and *in vivo* feeding experiment (Lopes et al., 2004). And there are indeed furan lignans (chicanine and d-epigalbacin) in *S. chinensis* (Liu et al., 1981; Hancke et al., 1999). Based on the existing research results, the comparison of chemical structures and the similarity of lignan biosynthesis, Suzuki et al. proposed the unique and authoritative biosynthetic pathway for dibenzocyclooctadiene lignans (Figure 1; Suzuki and Umezawa, 2007). What is more, Suzuki et al. also proposed that yatein was the precursor of deoxypodophyllotoxin in 2007 (Suzuki and Umezawa, 2007), which was proved to be right in 2015 (Lau and Sattely, 2015). In conclusion, we believe the biosynthetic pathway for dibenzocyclooctadiene lignans is also right and we are striving to clone functional genes according to this pathway.

As shown in Figure 1, coniferyl alcohol is synthesized from phenylalanine through the general phenylpropanoid pathway. It should be noted that coniferyl alcohol is not only a synthetic precursor of lignans but also a kind of monolignol involved in the biosynthesis of lignin (Vanholme et al., 2010). Therefore, the biochemical steps before coniferyl alcohol are shared between dibenzocyclooctadiene lignan and lignin biosynthesis (Muhlemann et al., 2014). Consequently, coniferyl alcohol acyltransferase (CFAT), the first enzyme after the branch point, must play a vital role in the synthesis of dibenzocyclooctadiene lignans. CFAT is a member of the BAHD acyltransferase family, which has two conserved motifs, HXXXD and DFGWG (D’Auria, 2006). The BAHD acyltransferases depending on acyl-CoA are responsible for the acylation of compounds, which can improve the stability and solubility of compounds, protect substances from enzymatic degradation, help plants resist diseases, and so on (Yu et al., 2009). Since St-Pierre et al. first coined the name BAHD based on the first four enzymes (BEAT, AHCT, HCBT, and DAT; St-Pierre and Luca, 2000), the BAHD superfamily is fast-growing and found to play important roles in the biosynthesis of a variety of secondary metabolites, such as *Catharanthus* alkaloid vindoline (St-Pierre et al., 1998), the *Papaver* alkaloid morphine (Grothe et al., 2001), the diterpenoid alkaloid Taxol (Walker and Croteau, 2000a,b; Walker et al., 2000), anthocyanins (Suzuki et al., 2001, 2002), and floral volatiles (Dudareva et al., 1998), as well as some phytoalexins (Chakravarthy et al., 2010). However, to date, only two CFAT genes, *PhCFAT* and *PmCFAT1*, have been characterized from *Petunia* × *hybrida* and *Prunus mume* (Dexter et al., 2007; Zhang et al., 2019). The gene encoding CFAT has not been identified from *S. chinensis*.

Although some progress has been made in the biosynthesis of several types of lignans, it has been stymied to elucidate the biosynthetic pathway of dibenzocyclooctadiene lignans because the biosynthetic genes, the genetic transformation system, and the whole genome of *S. chinensis* are unavailable. In this study, to determine whether there is a gene encoding CFAT in *S. chinensis*, we confirmed the presence of isoeugenol in *S. chinensis* by GC–MS for the first time. Then, a total of 37 *ScBAHD* genes were obtained in a transcriptome-wide identification. Phylogenetic, conversed motifs, and expression pattern analyses were performed to screen the putative gene encoding CFAT in *S. chinensis*. Finally, *ScBAHD1* was demonstrated to be *ScCFAT* through heterologous expression and *in vitro* enzymatic reaction. Furthermore, to clarify the molecular mechanism of ScCFAT, especially to improve its catalytic efficiency, bioinformatics (molecular modeling and docking simulations) were used in this work. In summary, we identified the first enzyme with catalytic activity from the Schisandraceae family, which is of guiding significance for fully revealing the biosynthetic pathway of dibenzocyclooctadiene lignans.

## Materials and Methods

### Plant Materials, Reagents, and Chemicals

The fruits of *S. chinensis* were collected in the Beijing medicinal plant garden at IMPLAD (Institute of Medicinal Plant Development; 40°N and 116°E), Beijing, China. The air-dried samples were pulverized into powder, passed through a 50-mesh sieve, and stored at room temperature until analysis. In addition, the green and fresh fruits of *S. chinensis* were immediately frozen in liquid nitrogen and stored at −80°C for RNA extraction. Ethyl acetate (UPLC grade) was purchased from Merck (Merck KGaA, Darmstadt, Germany). Reference compounds, isoeugenol was purchased from Chengdu DeSiTe Biological Technology (Chengdu, China), and cinnamyl acetate was purchased from Shanghai Macklin Biochemical (Shanghai, China).

### Identification of Isoeugenol in *Schisandra chinensis*

The powdered *S. chinensis* fruits (1.0000 g) were suspended in 50 ml of UPLC grade ethyl acetate in a 100 ml conical flask with a stopper and then was extracted in an ultrasonic bath for 60 min at room temperature. The extract was collected by filtering and the residue was washed with 5 ml of ethyl acetate three times. Then, the extract was vacuum evaporated to recover the solvent. Later the residue was dissolved in 10 ml of ethyl acetate and the sample solution was filtered through a 0.22 μm membrane filter into the vials for GC–MS analysis. The standard stock solution of isoeugenol was prepared by dissolving it in UPLC grade ethyl acetate and then diluted to the appropriate concentration for GC–MS analysis.

### Instrument Parameters

GC–MS analysis for identification of isoeugenol in the fruits of *S. chinensis* was performed using an Agilent Technologies 7890B gas chromatography instrument, combined with an Agilent 5977A MSD equipped with electron ionization (EI) and quadrupole analyzer, and an Agilent Chem Station data system. GC separation was performed on a 30 m HP-5MS Ultra Inert capillary column with an internal diameter of 0.25 mm and a film thickness of 0.25 μm (Agilent 19091S-433UI, Agilent Technologies). The carrier gas was helium (99.99%) with a flow rate of 1 ml/min. The temperature of the injector and detector was set at 280°C and 250°C, respectively. Spectra were obtained over a scan range of 50 to 550 amu at 2.9 scans/s. The GC program was set as follows: the initial temperature was 60°C and held for 2 min, then increased by 5°C/min to 135°C, then raised by 3°C/min to 180°C, and finally raised by 10°C/min to 200°C and held at 200°C for 10 min. The sample and isoeugenol solution (1 μl) were injected manually while maintaining a solvent delay of 4 min. Interpretation of the mass spectrum was made by comparing the peak distribution against the database of National Institute Standard and Technology (NIST MS 2.0, Gaithersburg, MD, USA).

### Identification and Bioinformatics Analyses of *ScBAHD* Genes

The transcriptome datasets (PRJNA533953) of *S. chinensis* (Chen et al., 2020b) were downloaded from NCBI^1^ and used for the identification of *ScBAHD* genes. The Hidden Markov Model (HMM) profile of the transferase domain (PF02458) downloaded from the Pfam database (Mistry et al., 2021) was exploited for the identification of *ScBAHD* genes by using the simple HMM search program of TBtools (Eddy, 2004; Chen et al., 2020a). The NCBI Batch Web CD-Search Tool with default parameters^2^ was exploited to test for the presence of the transferase domain. The sequence integrity of ScBAHDs was analyzed by performing multiple sequence alignment analyses of all ScBAHDs by ClustalW (Chenna et al., 2003). Some ScBAHDs, whose number of amino acids is shorter than 400 or longer than 500 were removed (D’Auria, 2006). ExPASy^3^ was used to calculate the length of protein sequences, molecular weight (M_W_), theoretical isoelectric point (pI), and the grand average of hydropathicity (GRAVY; Artimo et al., 2012). Wolf PSORT^4^ was employed to predict the subcellular localization of ScBAHD proteins (Horton et al., 2007).

### Conserved Motif, Alignment, and Phylogenetic Analyses

The peptide sequences of putative ScBAHD proteins were submitted to MEME 5.4^5^ for the analysis of conserved motif composition (Bailey et al., 2015). The optimized parameters were set as follows: the maximum number of motifs was 10, the width of each motif comprised between 6 to 50 residues, and the remaining parameters were default. Putative ScBAHD protein sequences were aligned along with 75 biochemically characterized BAHD members (Supplementary Table S1) using MAFFT 7.4 (Katoh and Standley, 2013). The maximum likelihood phylogenetic tree was constructed by IQ-TREE 2 with 1,000 bootstrap replicates (Minh et al., 2020). And the iTOL online tool^6^ was used to visual embellish (Letunic and Bork, 2021).

### Transcriptome Datasets Analysis

The transcriptome datasets of *S. chinensis* fruits at three stages during fruit ripening (green, light red, and dark red) obtained from NCBI (PRJNA533953; Chen et al., 2020b) were used for expression pattern analysis. The expression level of each gene at three stages was calculated by using Bowtie2 (Langmead and Salzberg, 2012) and RSEM (Li and Dewey, 2011), and converted into transcripts per million reads (TPM). The data were visualized using an R package heatmap.

### Total RNA Extraction and cDNA Synthesis

Total RNA was extracted from the fresh fruits of *S. chinensis* by using the EASYspin Plus Complex Plant RNA Kit (Aidlab Biotechnology, Beijing, China) following the manufacturer’s instructions. The quality of total RNA was verified by absorbance at 260 nm and 280 nm optical densities by using the NanoDrop 2000 Spectrophotometer (Thermo Scientific, San Jose, CA, USA) and by 1% agarose gel electrophoresis. The first-strand cDNA was synthesized by using the GoScript™ Reverse Transcription Kit (Promega, Beijing, China) according to the instructions. The cDNA was stored at −80°C for further gene clone and qPCR.

### Validation of RNAseq by RT-qPCR

The results of RNAseq were validated by RT-qPCR to see if there is a good correlation between the expression of *ScBAHD1* obtained by both techniques. After entering the fruiting period, we regularly picked the *S. chinensis* fruits with three biological replicates at four different developmental stages every 30 days. The samples were numbered as 1-1, 1-2, 1-3 (three replicates of the first period), 2-1, 2-2, 2-3 (three replicates of the second period), 3-1, 3-2, 3-3 (three replicates of the third period), and 4-1, 4-2, 4-3 (three replicates of the fourth period). Primers (ScBAHD1-qF and ScBAHD1-qR) designed by CLC Genomics Workbench are listed in Supplementary Table S2. The qPCR experiments were performed in triplicate using TB Green Premix Ex Taq (Takara Biotechnology, Dalian, China) in a Bio-Rad CFX96 Real-Time system. *ScGAPDH* was the reference gene, and the relative expression levels were calculated by the 2^–ΔΔCt^ method from Ct values.

### Cloning and Sequencing of *ScCFAT* Full-Length cDNA

The specific primers (ScCFAT-F and ScCFAT-R) in Supplementary Table S2 were designed by using CLC Genomics Workbench. The full-length *ScCFAT* cDNA was amplified in a 25 μl system including 1 μl cDNA, 0.5 μl forward and reverse primer, 5 μl 5× TransStart FastPfu Buffer, 2 μl 2.5 mM dNTPs, 15.5 μl ddH_2_O, and 0.5 μl TransStart FastPfu DNA Polymerase (TransGen Biotech, Beijing, China). The amplification procedure was shown as follows: 95°C for 1 min, 40 cycles of 95°C for 20 s, 55°C for 20 s, 72°C for 45 s, and a final extension step for 5 min at 72°C. Subsequently, the amplification products of proper length were purified by using Gel DNA Mini Purification Kit (Aidlab Biotechnology, Beijing, China) and ligated into the pET-28a vector digested by QuickCut™ *Eco*RI through seamless cloning by using ClonExpress Ultra One Step Cloning Kit (Vazyme, Nanjing, China). The recombinant plasmid was transformed into *Escherichia coli* DH5α competent cell and sequenced in Tsingke Biotechnology (Beijing, China). Then, the right plasmid was isolated and transformed into *E. coli* BL21 (DE3) competent cell.

### Heterologous Expression and Recombinant Protein Purification

*Escherichia coli* BL21 (DE3), which was transformed into the recombinant plasmid, was grown in LB medium with 50 μg/ml kanamycin at 37°C until the OD_600_ of 0.6–0.8 was reached. The expression was induced by adding 0.1–0.2 mM Isopropylb-D-1-thiogalactopyranoside (IPTG) at 18°C for 18 h. After induction, the cells were collected, resuspended in 1× PBS buffer, and broken by sonication. The lysate was centrifuged at 4°C and the supernatant was collected for purification by using Hiper Ni-Agarose His tag soluble protein purification kit (Mei5 Biotechnology, Beijing, China). After denaturing with SDS loading dye at 100°C for 5 min, the eluted fractions were examined through 12% SDS-PAGE gel electrophoresis followed by staining of the gel with Coomassie brilliant blue.

### Enzyme Activity Assays *in vitro*

The 200 μl reaction mixture contained 56 μl purified protein, 140 μM acetyl-CoA, and 120 μM alcohol substrate in assay buffer (50 mM citric acid, pH 6.0, 1 mM DTT or 50 mM Tris–HCL, pH 7.5, and 1 mM DTT). While in the control group, instead of purified protein, 56 μl protein of empty vector was added to the reaction mixture. After incubation in 25°C water bath for 15 min, added 200 μl of chromatographic grade methanol into the mixture and centrifugated at 13,000 rpm for 25 min. Then, 200 μl upper reaction mixture was absorbed into vials and the products were verified by using UPLC.

### Transient Expression and Subcellular Localization

The pCAMBIA1302 vector driven by the cauliflower mosaic virus (CaMV) 35S promoter was used to identify the subcellular localization of ScCFAT by utilizing the transient expression system of *Nicotiana benthamiana* leaves. The entire coding sequence (CDS) of ScCFAT without its stop codon was amplified with primers (ScCFAT-LF and ScCFAT-LR) listed in Supplementary Table S2. The pCAMBIA1302 vector was digested by *Spe*I (TaKaRa Biotechnology, Dalian, China). Then, the CDS of *ScCFAT* was inserted into the digested vector to generate ScCFAT-GFP fusion protein. The resulting construct harboring *ScCFAT*-GFP gene was transferred into *Agrobacterium tumefaciens* strains GV3101 through the conventional freezing–thawing method, and the GV3101 strains harboring pCAMBIA1302-*ScCFAT*-GFP or empty pCAMBIA1302-GFP were transiently infiltrated into 5-week-old *N. benthamiana* leaves. After infection for 48 h, the green fluorescence protein (GFP) expression was observed under the Nikon C2-ER laser scanning confocal microscope.

### Homology Modeling and Docking Statistics

The BLASTP search for the amino acid sequence of ScCFAT was performed to obtain the most suitable template in the Brookhaven Protein Data Bank (PDB).^7^ The homology model of ScCFAT structure was generated by using Modeller 10.2 software. Five models were generated and selected according to the DOPE (Discrete Optimized Protein Energy) score. The stereochemical property of the model was evaluated by PROCHECK (Laskowski et al., 1996) and VERIFY-3D (Lüthy et al., 1992). The docking of ScCFAT with its natural substrate acetyl CoA and coniferyl alcohol was simulated by using AutoDock, and the visualized results were drawn by PyMOL.

## Results

### Identification of Isoeugenol in *S. chinensis*

**Figure 2:**
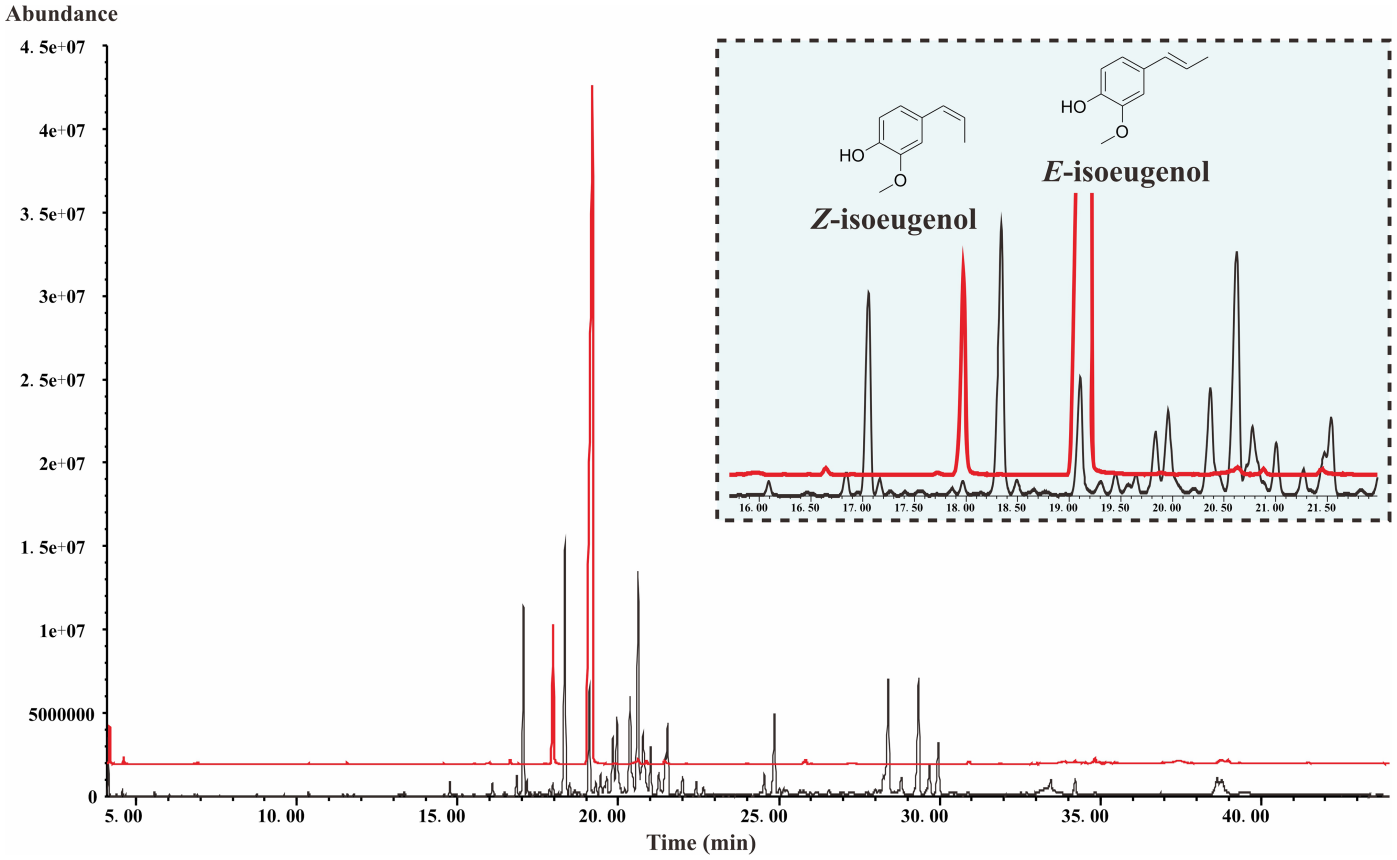
The TIC of the isoeugenol standard and the fruits of *S. chinensis*.

As shown in Figure 2, the total ion chromatogram (TIC) of the isoeugenol standard is presented in the red line and the TIC of the fruits of *S. chinensis* is presented in the black line. The mass profiles are presented in Supplementary Figure 1. The result indicates that *S. chinensis* contains both *E*-isoeugenol and *Z*-isoeugenol, and the former is dominant, which provides evidence that there must be a gene encoding CFAT in *S. chinensis*.

### Identification and Characteristics of *ScBAHD* Genes

The previous transcriptome datasets (PRJNA533953; Chen et al., 2020b) were used to identify all BAHD genes in *S. chinensis*. After removing short/long sequences and redundant sequences, we spotted 37 BAHD genes and named them *ScBAHD1* to *ScBAHD37*. As shown in Table 1, the length of 37 ScBAHD proteins varies from 425 to 489 amino acids, corresponding to M_w_ range of 43.40–52.66 kDa, the theoretical pI ranges from 4.68 to 8.38, and GRAVY varies from −0.310 to 0.076. Subcellular localization prediction results indicate that the majority of ScBAHD proteins (33 out of 37, 89%) localize in cytoplasm and chloroplast.

**Table 1:** Molecular characteristics of *ScBAHD* genes in *Schisandra chinensis*.

Gene ID	Gene name	CDS (bp)	Peptide (aa)	M_W_ (kDa)	pI	GRAVY	Subcellular localization
TRINITY_DN955_c1_g2_i8.p1	*ScBAHD1*	1,368	455	50.34	5.10	−0.162	Cytoplasm_Nucleur
TRINITY_DN62_c0_g1_i1.p1	*ScBAHD2*	1,302	433	47.93	6.96	−0.173	Chloroplast
TRINITY_DN955_c1_g2_i14.p1	*ScBAHD3*	1,446	481	50.28	5.48	−0.145	Cytoplasm
TRINITY_DN94461_c0_g1_i2.p1	*ScBAHD4*	1,347	448	50.09	5.00	−0.310	Cytoplasm
TRINITY_DN8709_c0_g3_i6.p1	*ScBAHD5*	1,368	455	51.01	6.26	−0.097	Chloroplast
TRINITY_DN8709_c0_g3_i3.p1	*ScBAHD6*	1,293	430	48.17	5.53	−0.123	Chloroplast
TRINITY_DN955_c1_g2_i19.p1	*ScBAHD7*	1,443	480	50.84	5.32	−0.138	Cytoplasm
TRINITY_DN5750_c1_g2_i1.p1	*ScBAHD8*	1,329	442	49.45	6.75	−0.143	Chloroplast
TRINITY_DN5598_c0_g1_i1.p1	*ScBAHD9*	1,407	468	52.66	6.20	−0.025	Chloroplast
TRINITY_DN5357_c1_g1_i6.p1	*ScBAHD10*	1,323	440	48.91	6.89	−0.002	Chloroplast
TRINITY_DN4048_c2_g1_i2.p1	*ScBAHD11*	1,293	430	47.55	8.20	−0.050	Chloroplast
TRINITY_DN3726_c0_g1_i12.p1	*ScBAHD12*	1,338	445	49.76	8.38	−0.067	Peroxisome
TRINITY_DN34303_c0_g1_i1.p1	*ScBAHD13*	1,353	450	50.37	6.61	−0.077	Chloroplast
TRINITY_DN3385_c0_g1_i2.p1	*ScBAHD14*	1,278	425	47.70	5.83	−0.091	Nuclear
TRINITY_DN3165_c0_g1_i1.p1	*ScBAHD15*	1,299	432	48.30	5.57	−0.108	Cytoplasm
TRINITY_DN2803_c0_g1_i13.p1	*ScBAHD16*	1,338	445	49.40	7.24	−0.017	Cytoplasm
TRINITY_DN2803_c0_g1_i10.p1	*ScBAHD17*	1,338	445	49.52	6.78	−0.077	Cytoplasm
TRINITY_DN2683_c4_g1_i1.p1	*ScBAHD18*	1,470	489	50.30	6.51	−0.162	Chloroplast
TRINITY_DN2670_c0_g1_i24.p1	*ScBAHD19*	1,332	443	49.13	6.74	−0.167	Chloroplast
TRINITY_DN2670_c0_g1_i2.p1	*ScBAHD20*	1,332	443	49.21	5.80	−0.123	Chloroplast
TRINITY_DN2647_c1_g1_i1.p1	*ScBAHD21*	1,299	432	48.03	7.72	−0.037	Chloroplast
TRINITY_DN225916_c0_g1_i1.p1	*ScBAHD22*	1,305	434	43.40	5.91	−0.217	Cytoplasm
TRINITY_DN217545_c0_g1_i1.p1	*ScBAHD23*	1,353	450	50.54	5.60	−0.058	Cytoplasm
TRINITY_DN1974_c0_g1_i5.p1	*ScBAHD24*	1,374	457	51.53	7.98	−0.241	Peroxisome
TRINITY_DN192399_c0_g1_i2.p1	*ScBAHD25*	1,365	454	47.55	4.68	−0.026	Chloroplast
TRINITY_DN17737_c1_g1_i3.p1	*ScBAHD26*	1,308	435	49.03	4.97	−0.157	Cytoplasm
TRINITY_DN1760_c0_g1_i6.p1	*ScBAHD27*	1,413	470	49.60	5.68	−0.153	Chloroplast
TRINITY_DN1760_c0_g1_i12.p1	*ScBAHD28*	1,407	468	49.83	5.86	−0.138	Chloroplast
TRINITY_DN17481_c0_g1_i1.p1	*ScBAHD29*	1,374	457	50.28	6.78	−0.040	Chloroplast
TRINITY_DN17405_c0_g1_i1.p1	*ScBAHD30*	1,326	441	48.37	4.79	0.001	Cytoplasm
TRINITY_DN1676_c0_g1_i8.p1	*ScBAHD31*	1,329	442	49.23	7.94	0.076	Cytoplasm
TRINITY_DN1676_c0_g1_i7.p1	*ScBAHD32*	1,332	443	49.42	5.37	0.020	Cytoplasm
TRINITY_DN1621_c1_g1_i1.p1	*ScBAHD33*	1,392	463	51.12	6.60	−0.121	Cytoplasm
TRINITY_DN15_c0_g1_i14.p1	*ScBAHD34*	1,284	427	47.61	7.42	−0.235	Cytoplasm
TRINITY_DN15037_c0_g1_i2.p1	*ScBAHD35*	1,389	462	48.87	6.29	−0.068	Nuclear
TRINITY_DN130037_c0_g1_i2.p1	*ScBAHD36*	1,368	455	49.83	5.12	−0.152	Cytoplasm
TRINITY_DN10987_c0_g1_i1.p1	*ScBAHD37*	1,317	438	48.88	6.61	−0.234	Cytoplasm

### Alignment, Phylogenetic, and Conserved Motif Analyses

**Figure 3:**
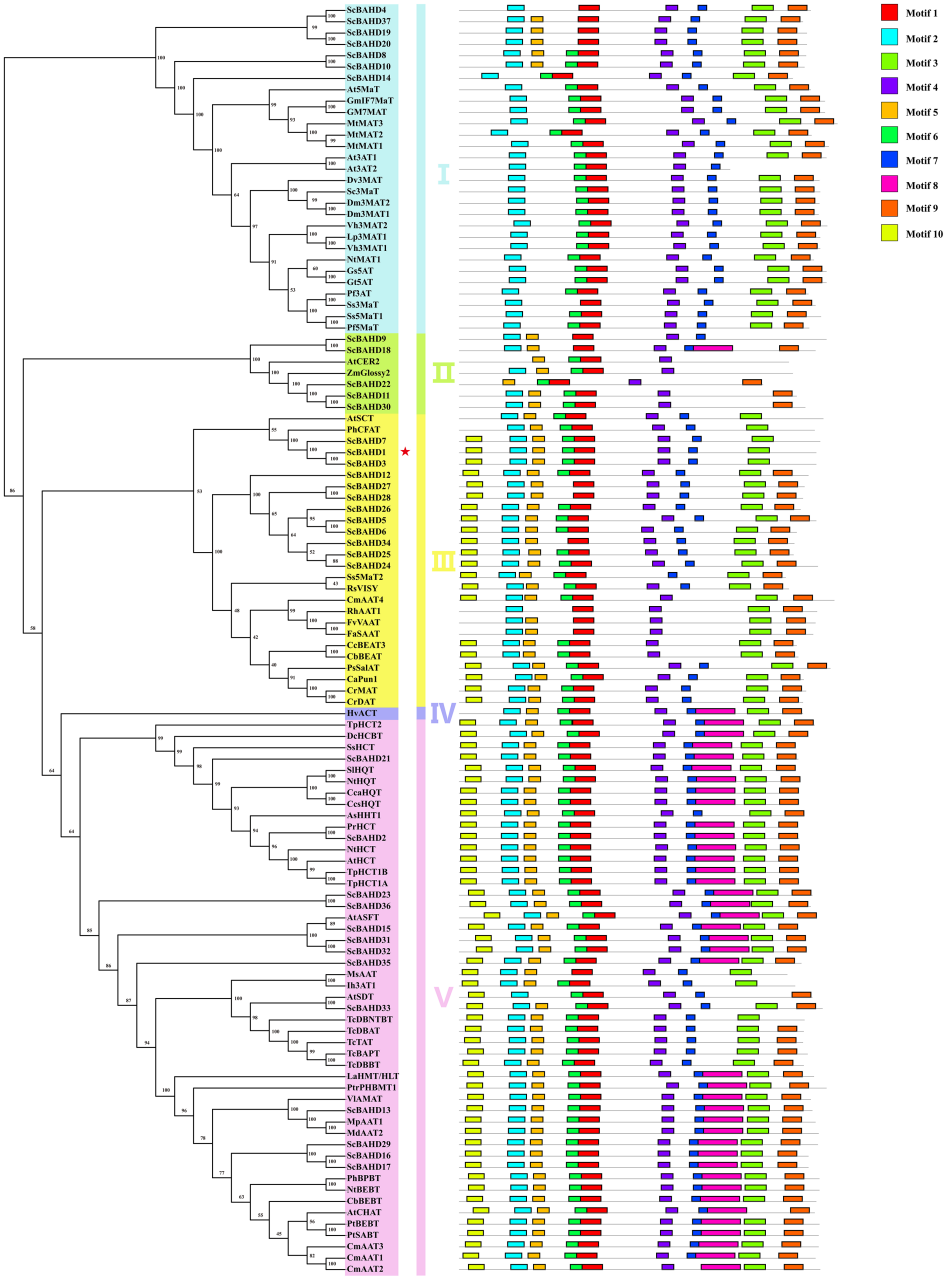
Maximum likelihood phylogenetic tree and conversed motif of 75 biochemically characterized BAHD acyltransferases and 37 putative ScBAHD acyltransferases. The five clades are shown in different colors.

Phylogenetic relationships among 75 biochemically characterized BAHD acyltransferases from other species (Supplementary Table S1) and 37 putative ScBAHD acyltransferases were constructed using a maximum likelihood algorithm. As shown in Figure 3, the phylogenetic tree is divided into five clades. There are seven ScBAHDs (ScBAHD4, ScBAHD8, ScBAHD10, ScBAHD14, ScBAHD19, ScBAHD20, and ScBAHD37) clustered into clade I. ScBAHD9, ScBAHD11, ScBAHD18, ScBAHD22, and ScBAHD30 are clustered into clade II along with ZmGlossy2 and AtCER2. There are 12 ScBAHDs clustered into clade III, which contains the PhCFAT. And ScBAHD1, ScBAHD3, and ScBAHD7 are close to PhCFAT, which indicates that the three ScBAHD proteins may be CFAT in *S. chinensis*. In addition, there is not a ScBAHD clustered into clade IV with HvACT. The remaining ScBAHD sequences are clustered into clade V. The result of conserved motifs analysis indicates that the BAHD proteins in the same clade have similar motifs. Motif 1 contains the HXXXD domain and motif 3 contains the DFGWG domain. All 37 ScBAHD proteins have the motif HXXXD, and aside from nine ScBAHDs, the others have the conserved motif DFGWG. Motifs 1–7 and motif 9 are widely distributed, but they do not exist in certain clades. For example, motif 3 exists in all other clades except clade II. Motif 8 is merely present in clade II, IV, and V. And motif 10 is only present in clade III and V.

### Expression Profiles of *ScBAHD* Genes in Different Stages

**Figure 4:**
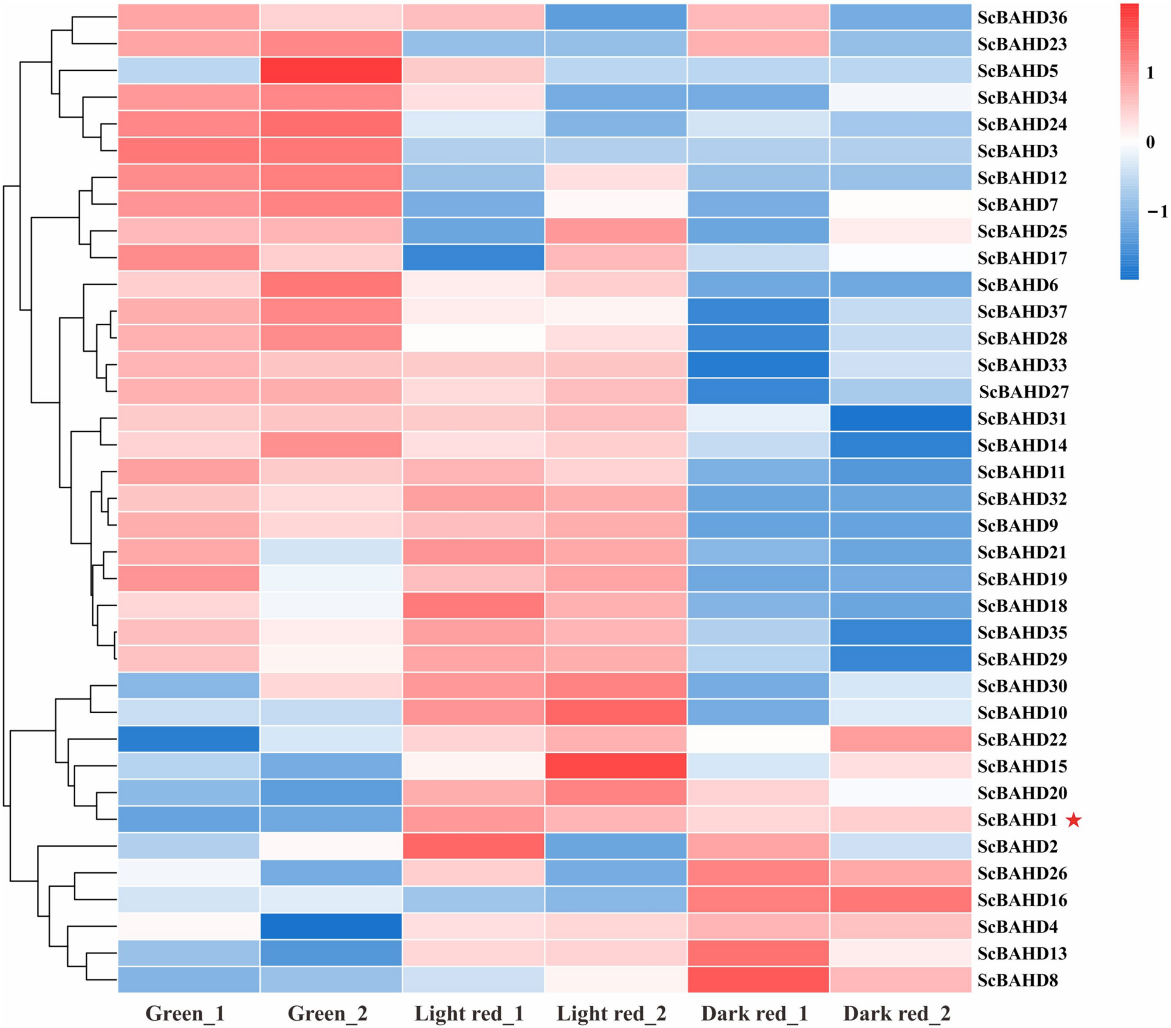
Heat map exhibits expression patterns of 37 *ScBAHD* genes during the ripening stages of *S. chinensis* fruits, including the green stage (Green_1 and Geen_2), light red stage (Light red_1 and Light red_2), and dark red stage (Dark red_1 and Dark red_2). Red indicates high relative gene expression, whereas blue indicates low relative gene expression.

To further investigate the expression profiles of 37 *ScBAHD* genes, we analyzed the expression levels of each *ScBAHD* gene during the ripening stages of the *S. chinensis* fruits, including the green stage, light red stage, and dark red stage. As shown in Figure 4, the clustering divides into three main clades. There are six *ScBAHD* genes (*ScBAHD3*, *ScBAHD5*, *ScBAHD23*, *ScBAHD24*, *ScBAHD34*, and *ScBAHD36*) primarily detected at the green stage. A total of 19 *ScBAHD* genes display higher expression at the green and light red stages than at the dark red stage. And 12 *ScBAHD* genes show high expression at the light red and dark red stages, but they are not detected at the green stage. *ScBAHD1* shows higher expression at the light red and dark red stages than at the green stage. However, *ScBAHD3* and *ScBAHD7* are not detected at the light red and dark red stages. The expression of *ScBAHD1* was further analyzed by RT-qPCR (Supplementary Figure 2), and the results were consistent with the RNAseq data.

### Heterologous Expression and Functional Characterization Analyses *in vitro*

**Figure 5:**
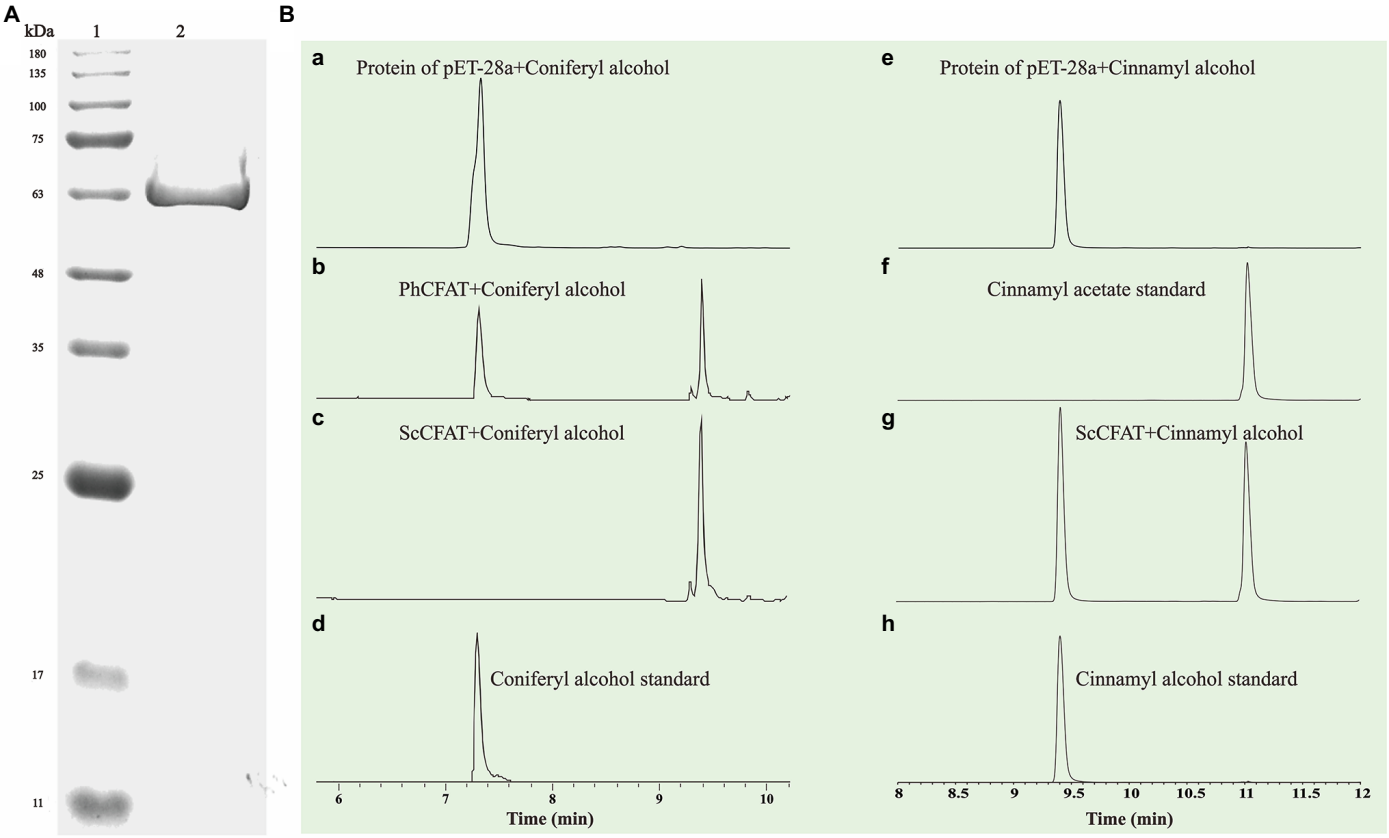
**(A)** SDS-PAGE analysis of recombinant ScCFAT. ScCFAT was separated using 12% SDS-PAGE gel electrophoresis and protein bands were visualized after staining with Coomassie brilliant blue. Lane 1: protein marker; Lane 2: purified ScCFAT. **(B)** *In vitro* analyses of ScCFAT and PhCFAT activities. (a) UPLC analysis of compounds found in a reaction mixture containing protein of pET-28a, coniferyl alcohol, and acetyl-CoA after 15 min of incubation. (b) UPLC analysis of compounds found in a reaction mixture containing purified PhCFAT, coniferyl alcohol, and acetyl-CoA after 15 min of incubation. (c) UPLC analysis of compounds found in a reaction mixture containing purified ScCFAT, coniferyl alcohol, and acetyl-CoA after 15 min of incubation. (d) UPLC analysis of coniferyl alcohol standard. (e) UPLC analysis of compounds found in a reaction mixture containing protein of pET-28a, cinnamyl alcohol, and acetyl-CoA after 15 min of incubation. (f) UPLC analysis of cinnamyl acetate standard. (g) UPLC analysis of compounds found in a reaction mixture containing purified ScCFAT, cinnamyl alcohol, and acetyl-CoA after 15 min of incubation. (h) UPLC analysis of cinnamyl alcohol standard.

*ScBAHD1*, named *ScCFAT* (1,368 bp, GenBank Accession Number, OM804184), encodes a protein of 455 amino acid residues, and the predicted molecular weight of ScCFAT is 50.34 kDa and the pI value is 5.10. *ScCFAT* was subcloned into the expression vector pET-28a using a restriction site to generate N-terminal hexa-histidine tagged recombinant proteins and the purified protein was assessed by SDS-PAGE (Figure 5A). The purified recombinant protein was evaluated for its ability to acetylate coniferyl alcohol as well as a variety of other alcohols by using acetyl-CoA as a source for the acetyl moiety. Assay products were analyzed by using UPLC. Because it has not been able to detect coniferyl acetate due to the instability (Koeduka et al., 2006), the product of ScCFAT catalyzing coniferyl alcohol was verified by using PhCFAT. As shown in Figure 5B, ScCFAT could catalyze coniferyl alcohol to the same product as PhCFAT catalyzed. In addition, ScCFAT could also catalyze cinnamyl alcohol into cinnamyl acetate (Figure 5B), which further proved the acetylation activity of ScCFAT. Apart from coniferyl alcohol and cinnamyl alcohol, ScCFAT exhibited detected activities with sinapyl alcohol, *p*-coumaryl alcohol, (*E*)-3-phenyl-2-methyl-2-propenol, benzyl alcohol, 4-hydroxybenzyl alcohol, and (+)-secoisolariciresinol as shown in Table 2 and Supplementary Figure 3.

**Table 2:** *In vitro* analyses of ScCFAT activities.

Substrate	CAS	Structure	Activity
Coniferyl alcohol	32811-40-8	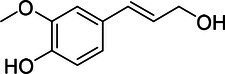	+
Cinnamyl alcohol	104-54-1	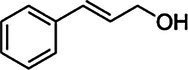	+
Sinapyl alcohol	537-33-7	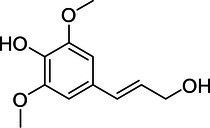	+
*p*-Coumaryl alcohol	3690-05-9	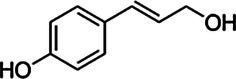	+
(*E*)-3-phenyl-2-methyl-2-propenol	1504-55-8	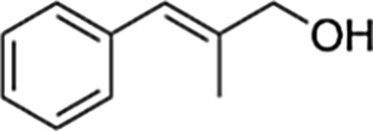	+
Isoeugenol	97-54-1	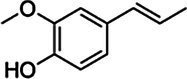	−
Eugenol	97-53-0	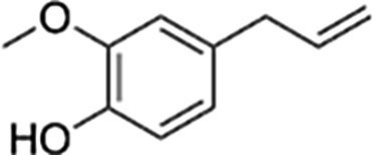	−
Ferulic acid	1135-24-6	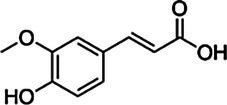	−
Gallic acid	149-91-7	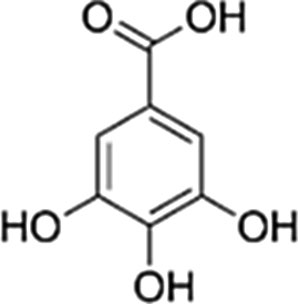	−
Benzyl alcohol	100-51-6	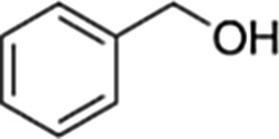	+
4-Hydroxybenzyl alcohol	623-05-2	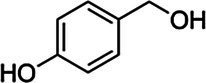	+
(+)-Secoisolariciresinol	145265-02-7	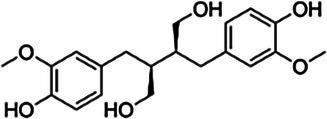	+
Quercetin	117-39-5	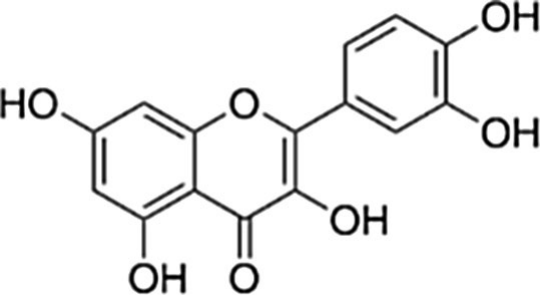	−
Kaempferol	520-18-3	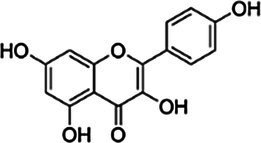	−
Liquiritigenin	578-86-9	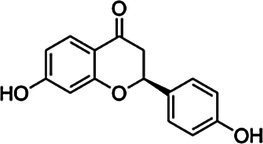	−
Isomucronulatol	52250-35-8	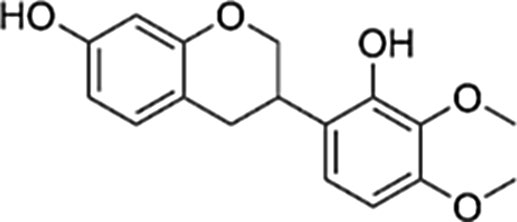	−
Myricetin	529-44-2	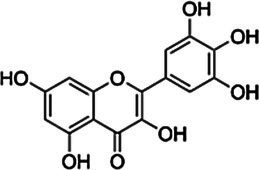	−
Naringenin	480-41-1	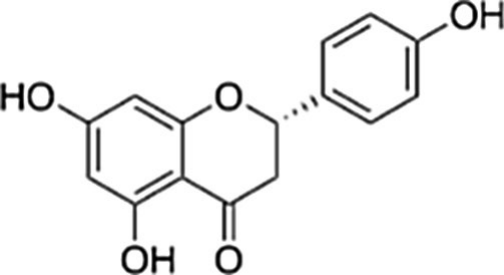	−

*“+” indicates ScCFAT can acetylate the corresponding substrate; “−” indicates ScCFAT cannot acetylate the corresponding substrate*.

### Subcellular Localization of ScCFAT

**Figure 6:**
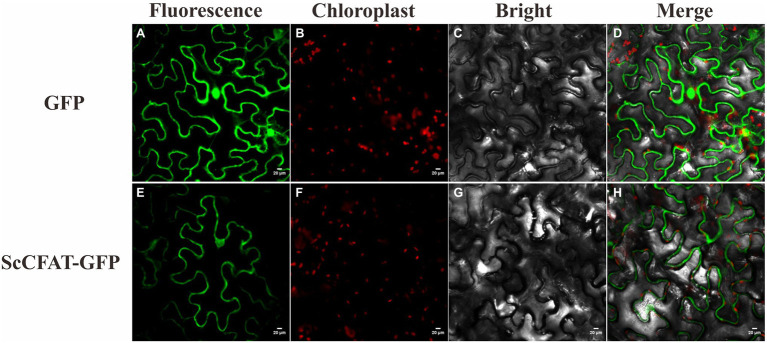
Subcellular localization of ScCFAT. The fusion proteins were observed under a confocal laser scanning microscope. a, e show the green fluorescence channel; b, f show the chloroplast autofluorescence channel; c, g show the bright field channel; and d, h were created from the images shown in the first three panels. Scale bar = 20 μm.

The prediction of protein subcellular localization with the WoLF PSORT Server showed that ScCFAT was located in the cytoplasm and nucleus with a probability of 26.32%. To investigate the intracellular localization of this protein, the nucleotide sequence without stop codon and with homologous sequences of restriction enzyme *Spe*I was obtained and ligated into the digested pCAMBIA1302 vector by homologous recombination. Transient transformation of *N. benthamiana* leaves was used to examine protein localization with the empty vector as a control. At 48 h after injection, the tobacco leaves were collected to observe the green fluorescence. As shown in Figure 6, the fluorescent signals of ScCFAT-GFP fusion protein are distributed predominantly within the cytoplasm of the cells. Therefore, ScCFAT appears to be primarily a cytoplasmic protein.

### Molecular Modeling and Docking of ScCFAT

Due to the divergent evolution of the BAHD family from one ancestral gene, this family displays low overall sequence identity (25–34%; St-Pierre and Luca, 2000), but these proteins possess highly similar three-dimensional (3D) structures (Walker et al., 2013). The best template for modeling the ScCFAT structure is the X-ray crystal structure of rosmarinic acid synthase from *Coleus blumei* (PDB accession number: 6MK2) with a sequence identity of 28.71%. The final unperturbed conformations of the model were acquired by progressively relaxing parts of the initial model. The stereochemical quality of the ScCFAT model was analyzed with PROCHECK. According to the Ramachandran plot, the most favored regions correspond to 81.7% of the structure, and only 1.8% of residues are placed in disallowed regions (Supplementary Figure 4). An analysis using Verify-3D shows that 81.32% of the residues have averaged 3D-1D score ≥ 0.2 (Supplementary Figure 5). The final structure of ScCFAT protein was accepted for subsequent analyses.

**Figure 7:**
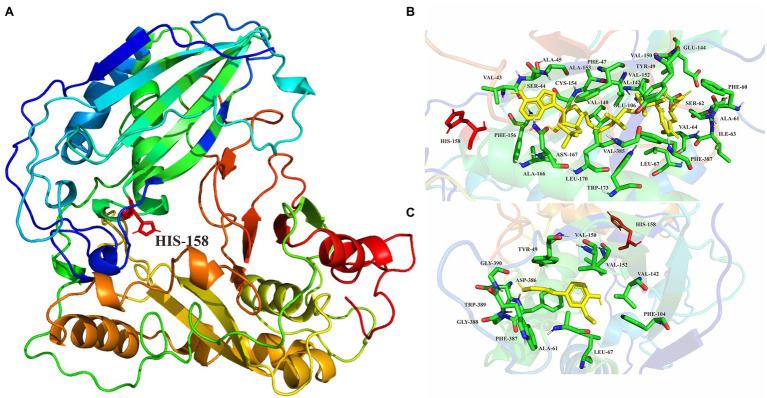
**(A)** Homology modeling of ScCFAT structure constructed with Modeller. His^158^ is shown in stick representation. **(B)** Enlarged views of molecular docking of ScCFAT with acetyl-CoA. **(C)** Enlarged views of molecular docking of ScCFAT with coniferyl alcohol.

The predicted ScCFAT structure consists of two nearly equal domains connected through a loop as shown in Figure 7A. A solvent channel runs through the ScCFAT molecule, allowing the substrate and co-substrate to bind independently. Results of docking simulation showed that the natural substrate acetyl-CoA was located in the solvent channel and docked with His^158^ as shown in Supplementary Figure 6. To identify the putative residues participating in the binding and/or catalysis of acetyl-CoA or coniferyl alcohol, molecular docking was conducted (Figures 7B,C). And the residues within 4 Å distance from the substrate position were selected as “hot spots” for the coming functional analysis.

## Discussion

### The Identification of Putative ScCFAT in *Schisandra chinensis*

CFAT, which must play an essential part in the biosynthetic pathway of dibenzocyclooctadiene lignans, is unknown in *S. chinensis*. Before studying *ScCFAT*, we first needed to confirm whether isoeugenol is produced in *S. chinensis* since the biosynthetic pathway of dibenzocyclooctadiene lignans is hypothetical and coniferyl acetate is unstable and undetected. However, no literature has confirmed the presence of isoeugenol in *S. chinensis*. Here, we report the extraction method, instrument parameters, and the first detection of isoeugenol in the fruits of *S. chinensis* by using GC–MS for the first time. The presence of isoeugenol not only proves that there must be a gene encoding CFAT in *S. chinensis*, but also indicates that the biosynthetic pathway of dibenzocyclooctadiene lignans shown in Figure 1 may be authentic.

PhCFAT, the first identified coniferyl alcohol acyltransferase from *Petunia* × *hybrida*, is critical to the production of isoeugenol and can catalyze the formation of coniferyl acetate from coniferyl alcohol and acetyl-CoA (Dexter et al., 2007). To pick out the *ScCFAT* gene, transcriptome-wide identification of BAHD genes in *S. chinensis* was performed and a total of 37 *ScBAHD* genes were obtained. Bioinformatics analyses were conducted and ScBAHDs were diverse based on their sequence and physiochemical properties. Then, we constructed a phylogenetic tree of the 37 putative ScBAHD proteins and 75 identified BAHD acyltransferases from other species. The phylogenetic tree is consistent with that of D’Auria (2006), who sorted 46 functionally characterized BAHD acyltransferases into five clades. The BAHD acyltransferases in clade I are mainly involved in the modification of anthocyanins (anthocyanin acyltransferases, AATs). Apart from HXXXD and DFGWG, most AATs share the sequence Tyr-Phe-Gly-Asn-Cys (YFGNC), which has been used as defining characteristic to clone new AATs by homology-based strategy (Nakayama et al., 2003). Although there are seven ScBAHDs clustered into clade I, only ScBAHD8, ScBAHD10, and ScBAHD14 have the motif YFGNC. Therefore, the three ScBAHDs are most likely to participate in the acylation of anthocyanins in *S. chinensis*. The BAHD acyltransferases in Clade III can acetylate a diverse range of alcohol substrates, and PhCFAT is clustered into this clade. ScBAHD1, ScBAHD3, and ScBAHD7 are close to PhCFAT, which shows that these three proteins might function as CFAT in *S. chinensis*. In addition, the BAHD proteins clustered into clade V can be subdivided into two well-supported groups corresponding to Tuominen’s clade Va and Vb (Tuominen et al., 2011).

Gene expression patterns can provide significant clues for predicting the biological function of genes. The content of dibenzocyclooctadiene lignans in the fruits of *S. chinensis* gradually increases as the fruit matures and reaches the maximum until the half-mature period (Zhao and Wang, 2011). To explore the expression profiles of 37 *ScBAHD* genes, the transcriptome datasets of *S. chinensis* fruits at the green, light red, and dark red stages were employed. The results indicate that the expression profile of *ScBAHD1* showed higher expression at the light red and dark red stages than at the green stage, which was consistent with the accumulation of dibenzocyclooctadiene lignans. Therefore, based on the phylogenetic analysis and expression profile, it is suggested that ScBAHD1 is most likely to function as ScCFAT involved in the biosynthesis of dibenzocyclooctadiene lignans in *S. chinensis*.

### ScCFAT Functions as an Alcohol Hydroxyl Acetyltransferase *in vitro*

The recombinant protein of ScBAHD1 was incubated with acetyl-CoA and coniferyl alcohol to assay its activity. Because coniferyl acetate is unstable, the product was detected by UPLC and verified with PhCFAT as a control. Functional analysis *in vitro* confirmed that ScBAHD1 catalyzed coniferyl alcohol to the same product as PhCFAT catalyzed, which indicated the ScBAHD1 we cloned from the *S. chinensis* fruits is ScCFAT. Subsequently, we screened the candidate genes coding IGS1 in *S. chinensis* through bioinformatics analyses and will conduct functional studies. PhCFAT is most efficient with coniferyl alcohol among the alcohol substrates tested and involved in the isoeugenol biosynthesis through RNAi-induced gene silencing (Dexter et al., 2007). From the size of the substrate peak area observed by UPLC (Figure 5B), we preliminarily found ScCFAT may have a higher activity with coniferyl alcohol than PhCFAT.

To further test the acetylation activity of ScCFAT, cinnamyl alcohol was used as an acceptor substrate with acetyl-CoA as a thioester donor. The product was verified to be cinnamyl acetate by using the authentic standard based on the same retention time. The ScCFAT enzyme was then further tested with various alcohol acceptors in combination with acetyl-CoA. Besides coniferyl alcohol and cinnamyl alcohol, ScCFAT exhibited detectable activity on sinapyl alcohol, *p*-coumaryl alcohol, (*E*)-3-phenyl-2-methyl-2-propenol, benzyl alcohol, 4-hydroxybenzyl alcohol, and (+)-secoisolariciresinol, but no activity was monitored for other tested substances. These results indicate that ScCFAT functionally prefers alcohol hydroxyl acetylation rather than phenol hydroxyl. Anyway, to determine whether coniferyl alcohol acts as the substate of ScCFAT *in vivo*, it is necessary to observe if there will be a decrease in the synthesis of isoeugenol after the suppression of the expression of *ScCFAT via* RNAi-induced gene silencing. However, at present, we lack an effective genetic transformation system of *S. chinensis*, which is time-consuming to establish. But we are working actively to carry out this part of work. In addition, the result of subcellular localization shows that ScCFAT is predominantly located in the cytoplasm, which is consistent with the prediction of BAHD acyltransferases as cytosolic soluble enzymes due to the lack of transit peptides or other sequences leading to localization in organelles or secretion (D’Auria, 2006).

Ma et al. crystallized and solved the X-ray crystal structure of vinorine synthase, which is the first representative of the BAHD superfamily (Ma et al., 2005). The structural analyses combined with biochemical and mutagenesis studies demonstrate that the HXXXD motif is involved in catalysis at the active site and is indispensable for acyltransferase activity, and the DFGWG motif is unique for BAHD enzymes and plays an important structural role (Suzuki et al., 2003; Bayer et al., 2004; Ma et al., 2005). Here, we generated a 3D structure of ScCFAT with the structure of *C. blumei* RAS as the template, thereby obtaining a reasonable model with 81.7% of the structure in the most favored regions. After molecular docking, the points within 4 Å distance from acetyl-CoA and coniferyl alcohol are selected as “hot spots” for individual mutations of the residues to L-alanine to identify putative residues participating in the binding and/or catalysis. Furthermore, site-directed mutagenesis and saturation mutagenesis of ScCFAT can be used to improve its catalytic efficiency against coniferyl alcohol, which potentially improves the yield of dibenzocyclooctadiene lignans in *S. chinensis*.

In addition, to determine the apparent optimal conditions for the enzymatic activity of ScCFAT, reactions need to be conducted at different temperatures, pH, and reaction times. Then, under the determined apparent optimal conditions, besides acetyl-CoA, the thioesters of malonyl-, benzoyl-, *p*-hydroxybenzoyl-, *p*-coumaroyl-, and feruloyl-CoAs are also needed to test as the potential substrates. We learned by consulting pieces of literature that the enzyme activity assay of CFAT should be measured by determining how much of the ^14^C-labeled acetyl group of acetyl-CoA is transferred to the side chain of acceptor substrate and the radioactive product is quantified through liquid scintillation counting (Dexter et al., 2007; Carlos et al., 2016; Zhao et al., 2021). We will further study the relative activities and the kinetics parameters of ScCFAT, crystallize and solve its X-ray crystal structure, and conduct a comprehensive study on its characteristics in combination with biochemical and mutagenic studies.

### ScCFAT Is Critical to the Biosynthetic Pathway of Dibenzocyclooctadiene Lignans

*S. chinensis* is indispensable as the source of dibenzocyclooctadiene lignans for medicines, food supplements, and cosmetics, but the *Schisandra* plants grow slowly. Chemical synthesis of dibenzocyclooctadiene lignans is feasible in theory, but it is multistage and in low overall yields (Chang et al., 2005). The core and unique features of dibenzocyclooctadiene lignans are biphenyl moiety and cyclooctadiene ring, which have long been regarded as interesting and challenging synthetic targets by organic chemists. The plant biotechnology method of *in vitro* culture of *S. chinensis* has potential usefulness. However, the amounts of dibenzocyclooctadiene lignans *in vitro* cultures were lower than that in the fruits (Szopa et al., 2016). According to biogenesis theory, biosynthesis is the most effective process in terms of energy consumption and yield. In recent years, the types and throughput of plant natural products synthesized by microorganisms are increasing, such as artemisinin (Paddon et al., 2013), tanshinones (Zhou et al., 2012; Guo et al., 2013), aglycons of ginsenosides (Dai et al., 2014), gastrodin (Bai et al., 2016), and so on.

Because the plant grows slowly, methods for constructing mutants and transgenic experiments are laborious, the discovery of biosynthetic genes involved in the biosynthesis of dibenzocyclooctadiene lignans is pioneering and challenging. Although Suzuki et al. proposed the biosynthetic pathway for dibenzocyclooctadiene lignans back in 2007, there has been no progress on functionally characterized enzymes of *S. chinensis* apart from ScDIR (GenBank accession number: ADR30610; Kim et al., 2012). The current research focus of our group is to decipher the pathway of dibenzocyclooctadiene lignans and we speculated on the enzymes involved in the four major uncharacterized steps shown in Figure 1. First, we consider that isoeugenol is converted to verrucosin under the action of an auxiliary oxidase and dirigent protein (DIR; Halls et al., 2004). And we identified DIR gene family in *S. chinensis* and screened candidate genes (Dong et al., 2021). Verrucosin is converted to dihydroguaiaretic acid under the action of pinoresinol/lariciresinol reductase (PLR; Katayama et al., 1992a,b). Then, dihydroguaiaretic acid is likely to be catalyzed by CYP450 and *O*-methyltransferases (OMT) to form pregomisin (Lau and Sattely, 2015; Nett et al., 2020). It is CYP450 catalyzing pregomisin to gomisin J (Ikezawa et al., 2008; Gesell et al., 2009).

Because ScCFAT is the first known and committed enzyme in the biosynthetic pathway of dibenzocyclooctadiene lignans, the subject of this study is to identify and obtain the sequence information of *ScCFAT*, which can be used to screen candidate genes coding DIR, PLR, CYP450, and OMT *via* co-expression analysis combined with gene family analyses. Therefore, ScCFAT is critical to the elucidation of the biosynthetic pathway of dibenzocyclooctadiene lignans. Subsequently, we will imitate the approach of podophyllotoxin biosynthetic pathway elucidation and use *Agrobacterium-*mediated transient expression in *N. benthamiana* to test the candidate genes (Lau and Sattely, 2015).

## Data Availability Statement

The sequence of ScCFAT reported in this article has been deposited in NCBI GenBank with accession number OM804184.

## Author Contributions

H-TL, B-GZ, and J-SL conceived and designed the study. T-YQ, X-LM, and YC carried out the experiments. T-YQ wrote the manuscript. Y-QD performed the bioinformatic analyses. H-ML and J-SL revised the manuscript. T-YQ, Y-QD, and J-SL analyzed the data. All authors contributed to the article and approved the submitted version.

## Funding

This research was funded by the Ability Establishment of Sustainable Use for Valuable Chinese Medicine Resources (No. 2060302-2002-05), the National Natural Science Foundation of China (No. 81872965), and the CAMS Initiative for Innovative Medicine (No. 2021-1-I2M-031).

## Conflict of Interest

The authors declare that the research was conducted in the absence of any commercial or financial relationships that could be construed as a potential conflict of interest.

## Publisher’s Note

All claims expressed in this article are solely those of the authors and do not necessarily represent those of their affiliated organizations, or those of the publisher, the editors and the reviewers. Any product that may be evaluated in this article, or claim that may be made by its manufacturer, is not guaranteed or endorsed by the publisher.

## Footnotes

^1^ https://www.ncbi.nlm.nih.gov/

^2^ https://www.ncbi.nlm.nih.gov/Structure/bwrpsb/bwrpsb.cgi

^3^ https://web.expasy.org/protparam/

^4^ https://wolfpsort.hgc.jp/

^5^ http://memesuite.org/tools/meme

^6^ https://itol.embl.de/

^7^ https://www.rcsb.org/

## Supplementary Material

Supplementary Figure 1 The mass profiles of the *S. chinensis* fruits and the isoeugenol standard.

Supplementary Figure 2 Gene expression analysis of *ScBAHD1* by RT-qPCR at different fruit developmental stages.

Supplementary Figure 3 UPLC analysis of compounds found in a reaction mixture containing purified PhCFAT, alcohol acceptors, and acetyl-CoA after 15 min of incubation.

Supplementary Figure 4 Evaluation of the predicted structure of ScCFAT by Ramachandran plot.

Supplementary Figure 5 Verify-3D graph drawn for the ScCFAT model.

Supplementary Figure 6 Molecular docking of substrate acetyl-CoA with modeled ScCFAT structure in the active site pocket.
