# Supplementary figures and images for: Identification, Molecular Cloning, and Functional Characterization of a Coniferyl Alcohol Acyltransferase Involved in the Biosynthesis of Dibenzocyclooctadiene Lignans in Schisandra chinensis
Source: Front Plant Sci. 2022 Jun 23;13:881342. doi: 10.3389/fpls.2022.881342 (PMC9260284; doi:10.3389/fpls.2022.881342)

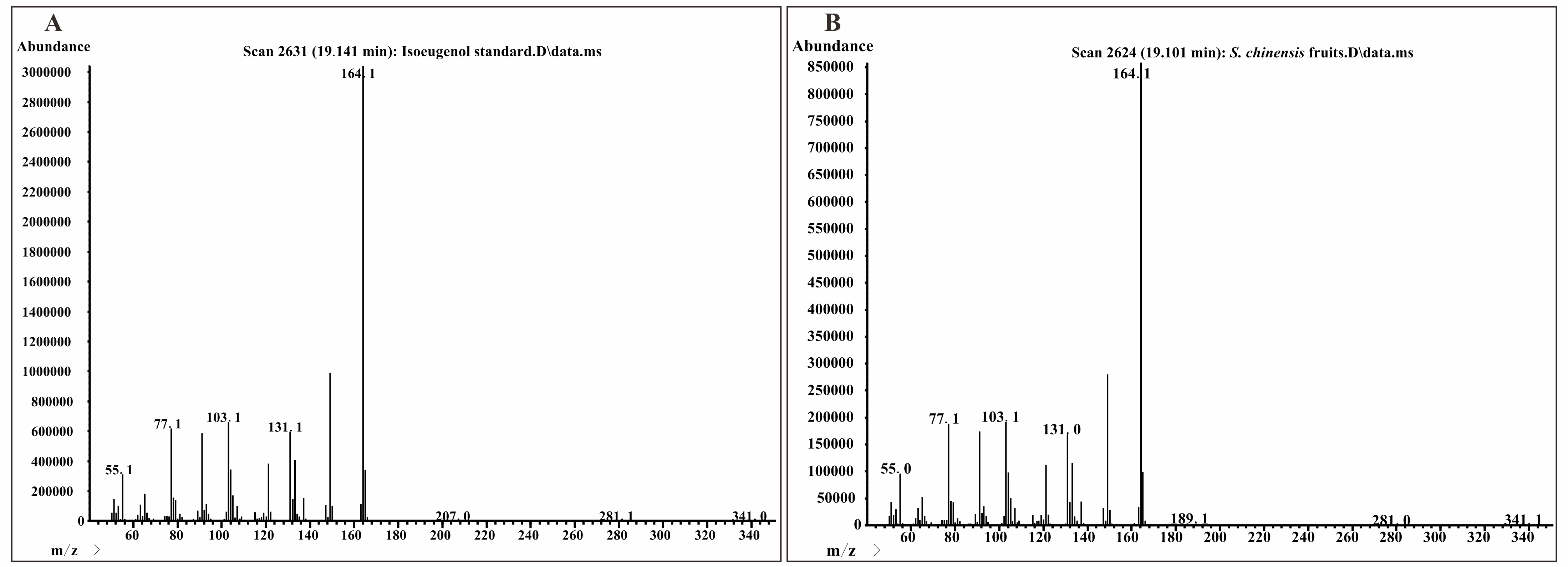

Supplement: Supplementary Figure 1 — The mass profiles of the S. chinensis fruits and the isoeugenol standard. [file Image_1.JPEG]

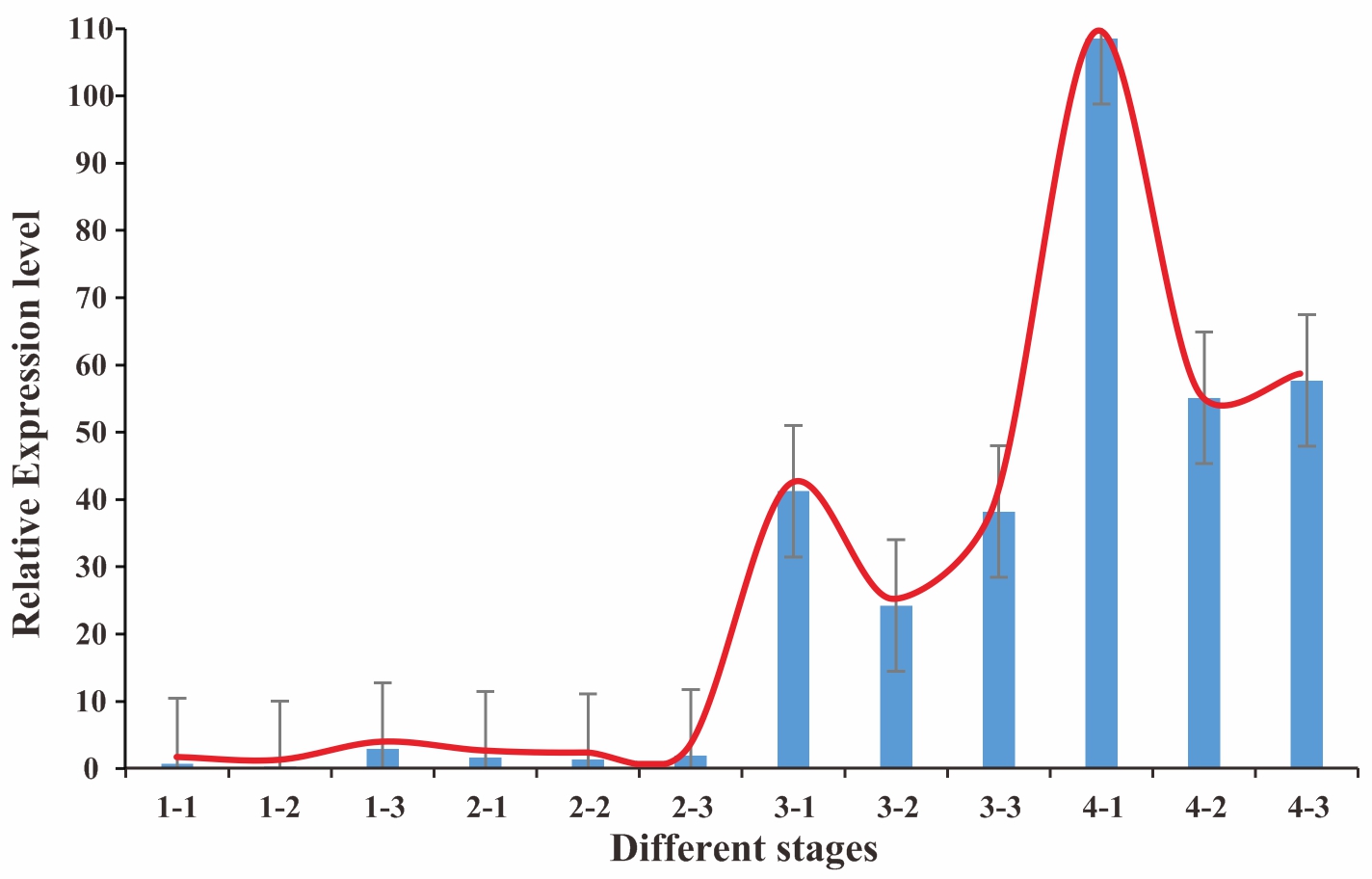

Supplement: Supplementary Figure 2 — Gene expression analysis of ScBAHD1 by RT-qPCR at different fruit developmental stages. [file Image_2.JPEG]

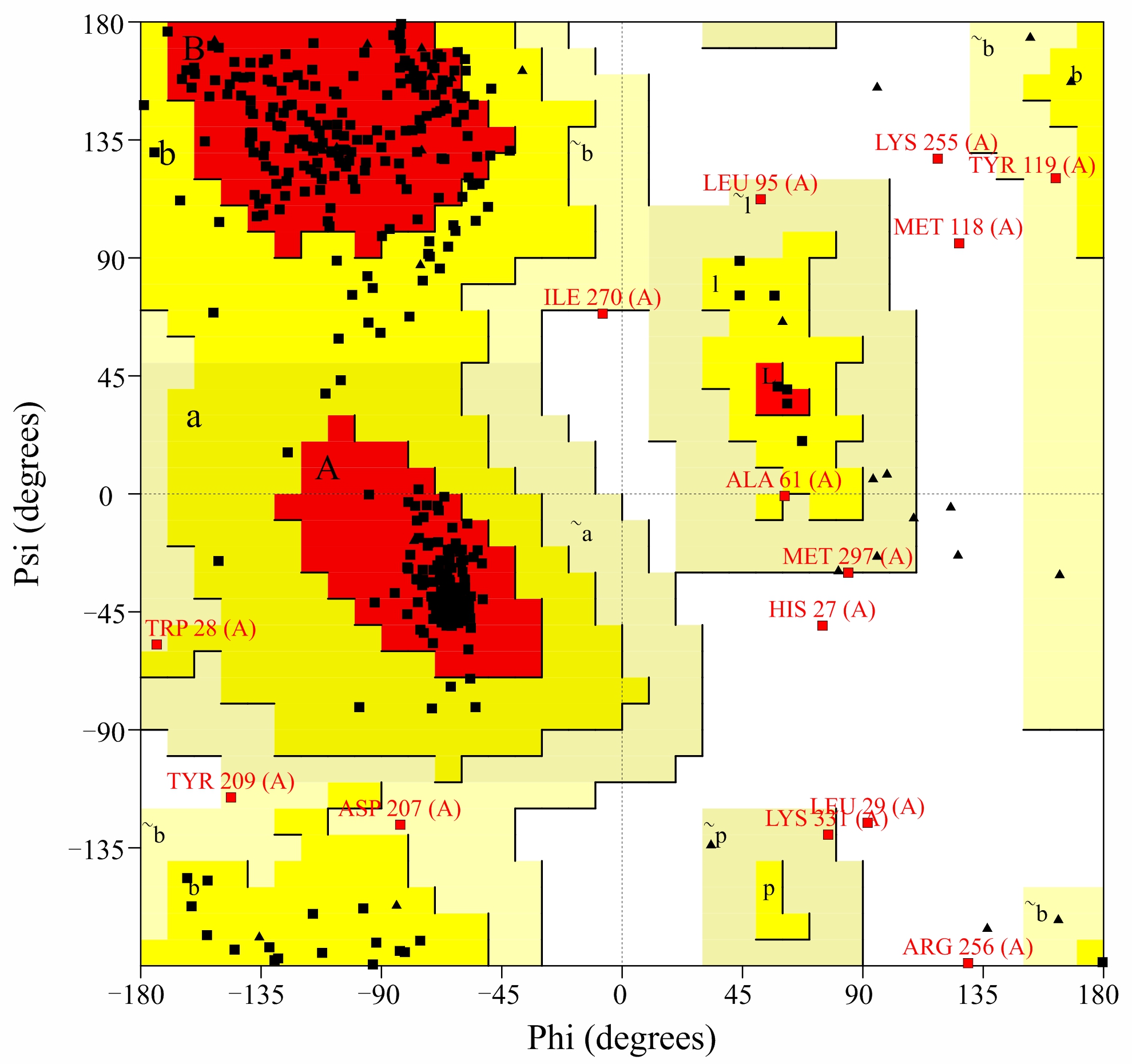

Supplement: Supplementary Figure 3 — UPLC analysis of compounds found in a reaction mixture containing purified PhCFAT, alcohol acceptors, and acetyl-CoA after 15 min of incubation. [file Image_3.JPEG]

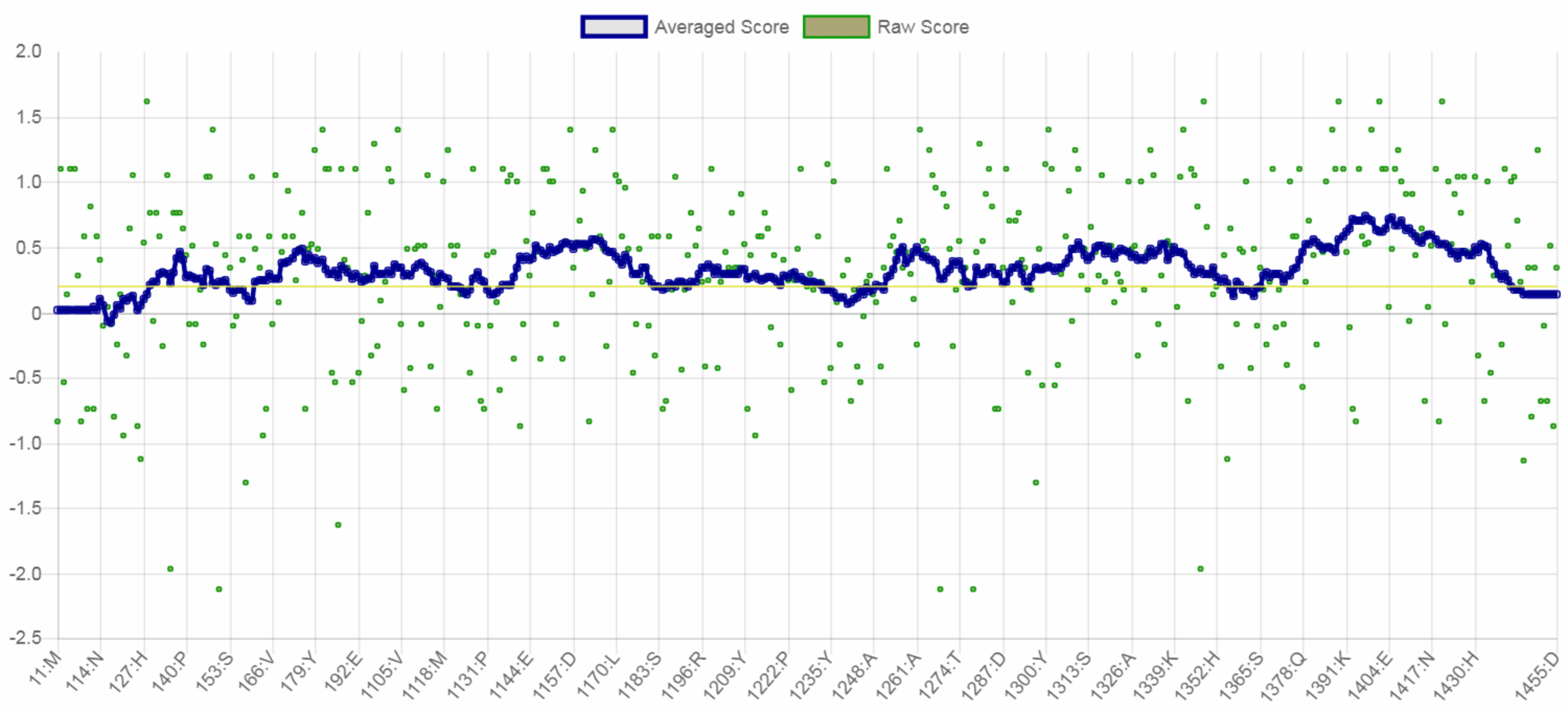

Supplement: Supplementary Figure 4 — Evaluation of the predicted structure of ScCFAT by Ramachandran plot. [file Image_4.JPEG]

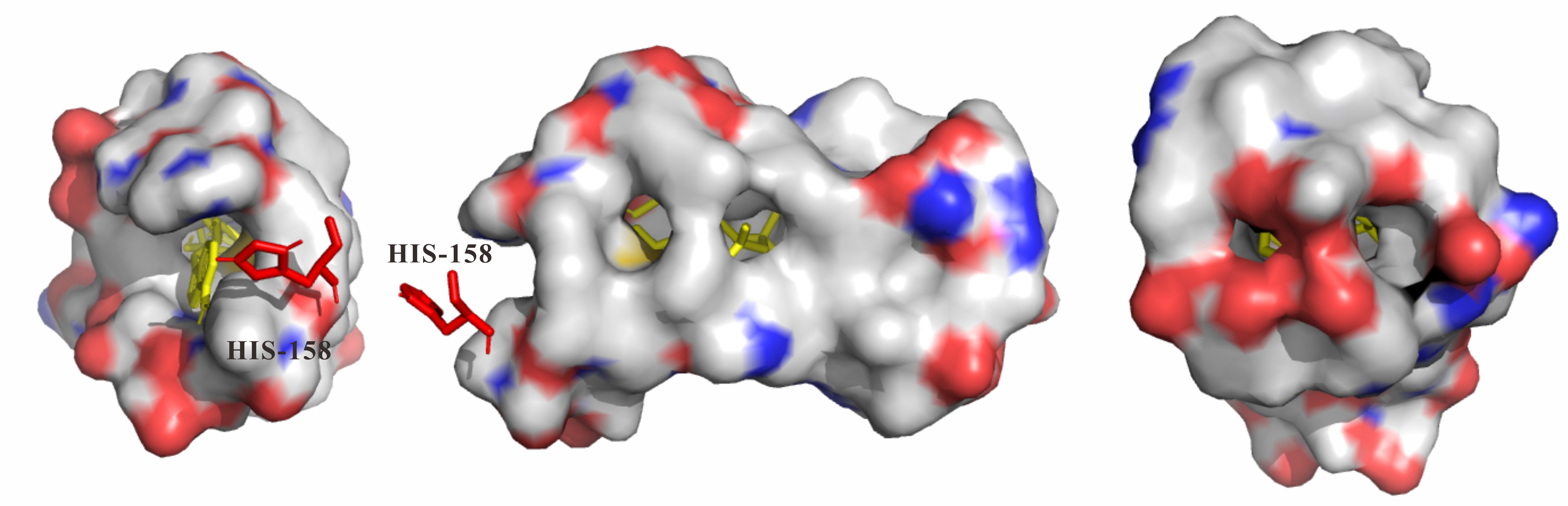

Supplement: Supplementary Figure 5 — Verify-3D graph drawn for the ScCFAT model. [file Image_5.JPEG]
