# Supplementary material for: Identification, Molecular Cloning, and Functional Characterization of a Coniferyl Alcohol Acyltransferase Involved in the Biosynthesis of Dibenzocyclooctadiene Lignans in Schisandra chinensis
Source: Front Plant Sci. 2022 Jun 23;13:881342. doi: 10.3389/fpls.2022.881342 (PMC9260284; doi:10.3389/fpls.2022.881342)
Supplement: Supplementary Figure 6 — Molecular docking of substrate acetyl-CoA with modeled ScCFAT structure in the active site pocket. [file Presentation_1.PPTX]

## Slide 1
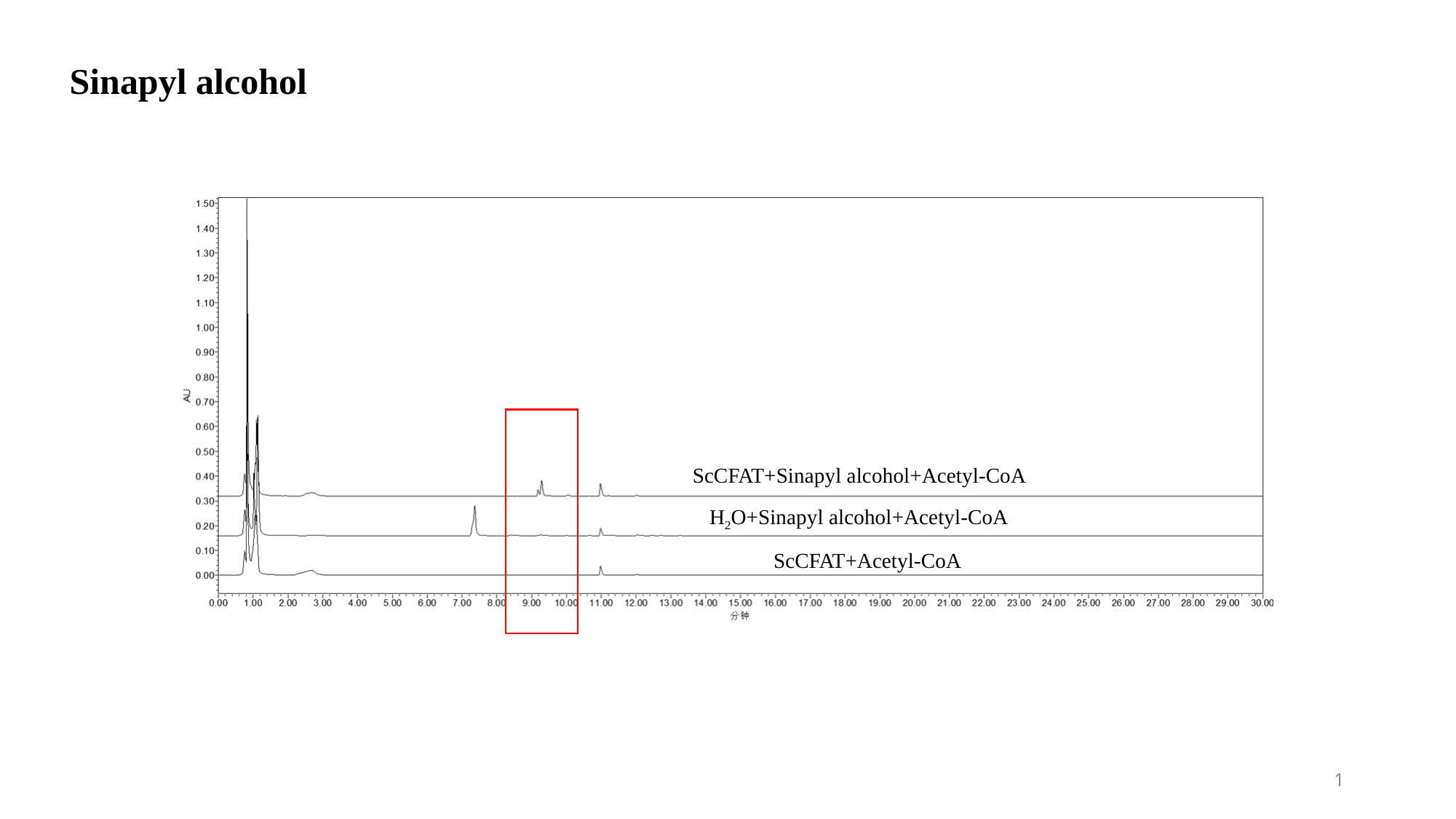

Sinapyl alcohol
ScCFAT+Sinapyl alcohol+Acetyl-CoA
H2O+Sinapyl alcohol+Acetyl-CoA
ScCFAT+Acetyl-CoA
1

## Slide 2
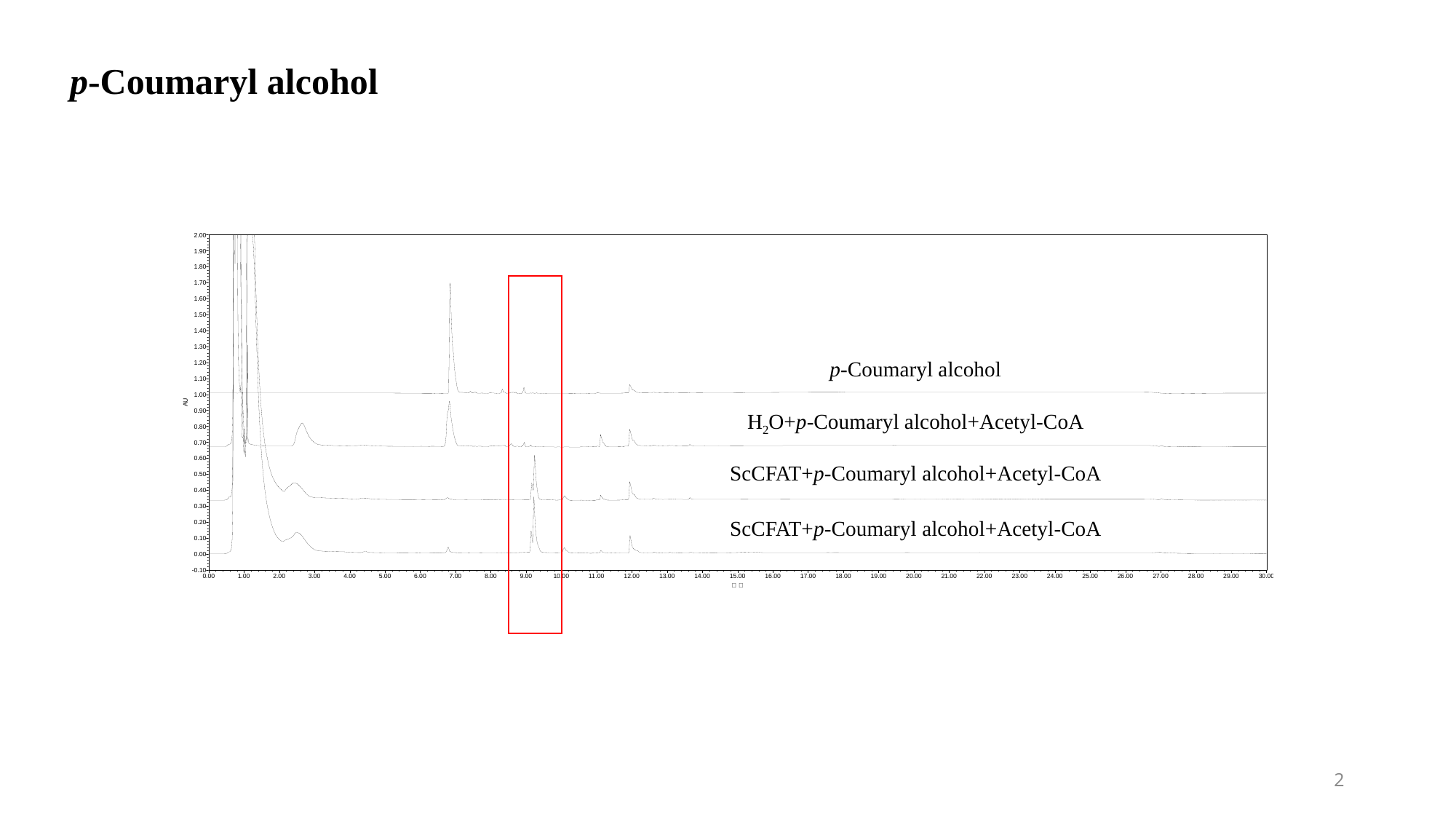

p-Coumaryl alcohol
p-Coumaryl alcohol
H2O+p-Coumaryl alcohol+Acetyl-CoA
ScCFAT+p-Coumaryl alcohol+Acetyl-CoA
ScCFAT+p-Coumaryl alcohol+Acetyl-CoA
2

## Slide 3
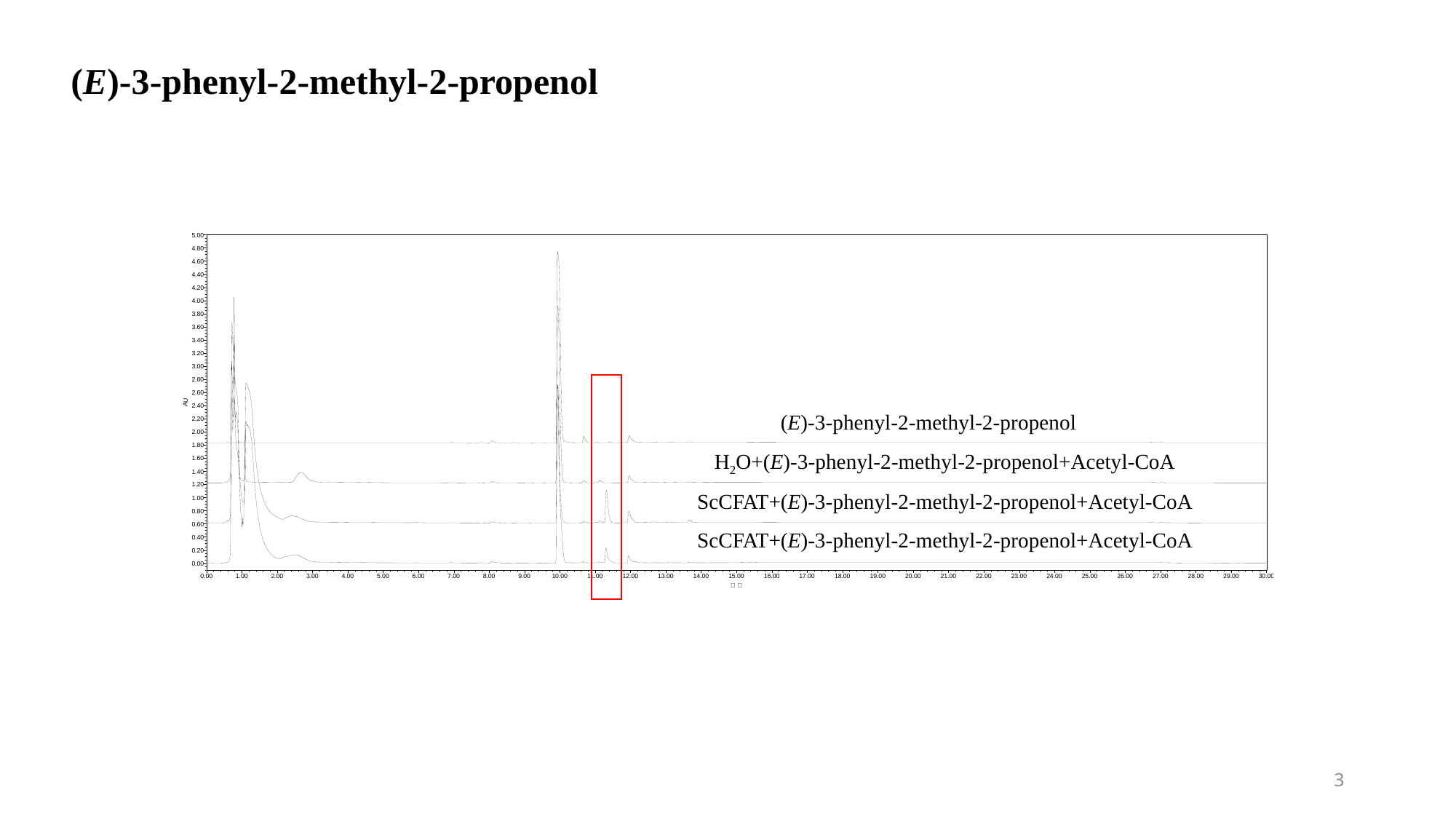

(E)-3-phenyl-2-methyl-2-propenol
(E)-3-phenyl-2-methyl-2-propenol
H2O+(E)-3-phenyl-2-methyl-2-propenol+Acetyl-CoA
ScCFAT+(E)-3-phenyl-2-methyl-2-propenol+Acetyl-CoA
ScCFAT+(E)-3-phenyl-2-methyl-2-propenol+Acetyl-CoA
3

## Slide 4
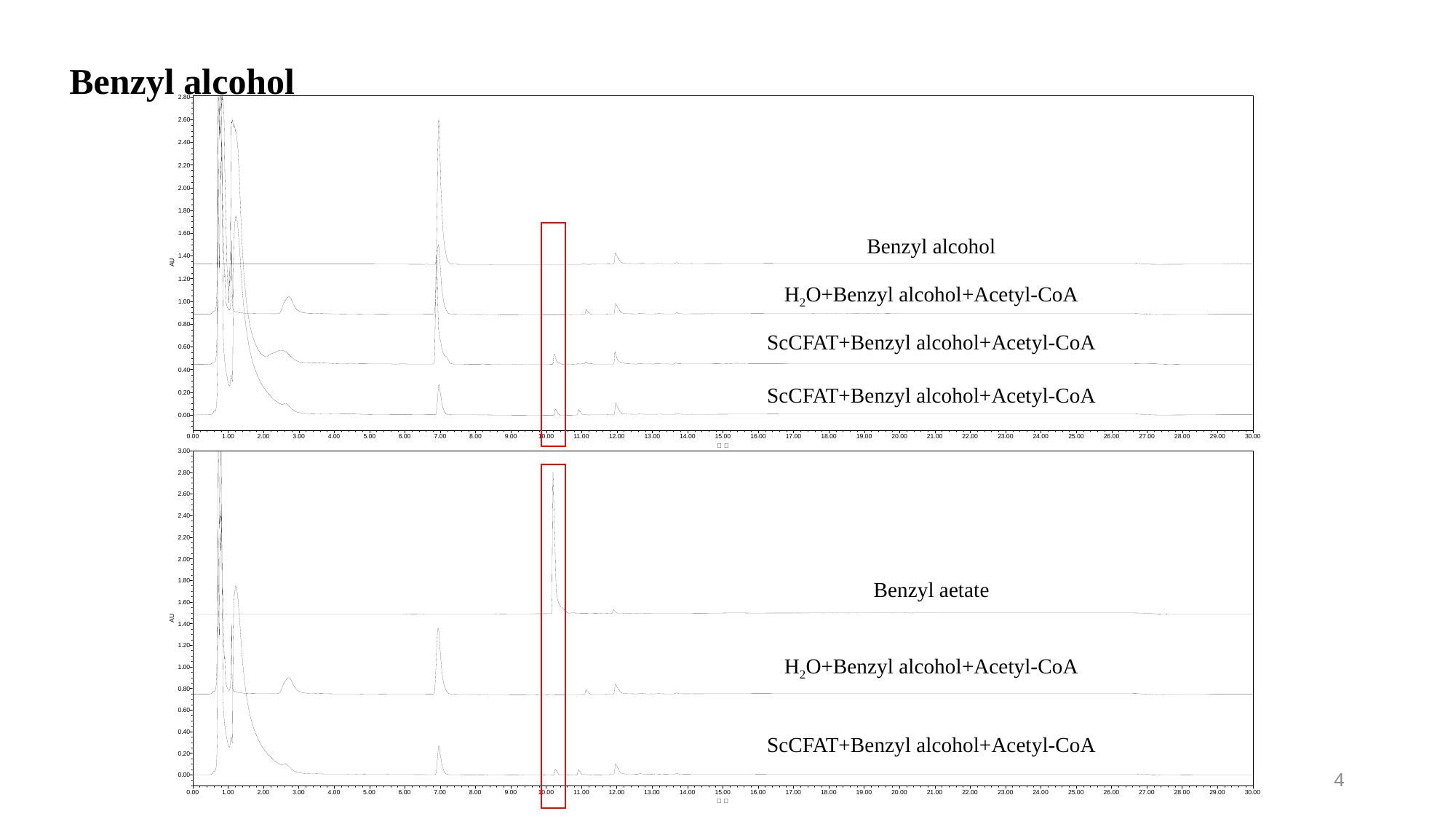

Benzyl alcohol
Benzyl alcohol
H2O+Benzyl alcohol+Acetyl-CoA
ScCFAT+Benzyl alcohol+Acetyl-CoA
ScCFAT+Benzyl alcohol+Acetyl-CoA
Benzyl aetate
H2O+Benzyl alcohol+Acetyl-CoA
ScCFAT+Benzyl alcohol+Acetyl-CoA
4

## Slide 5
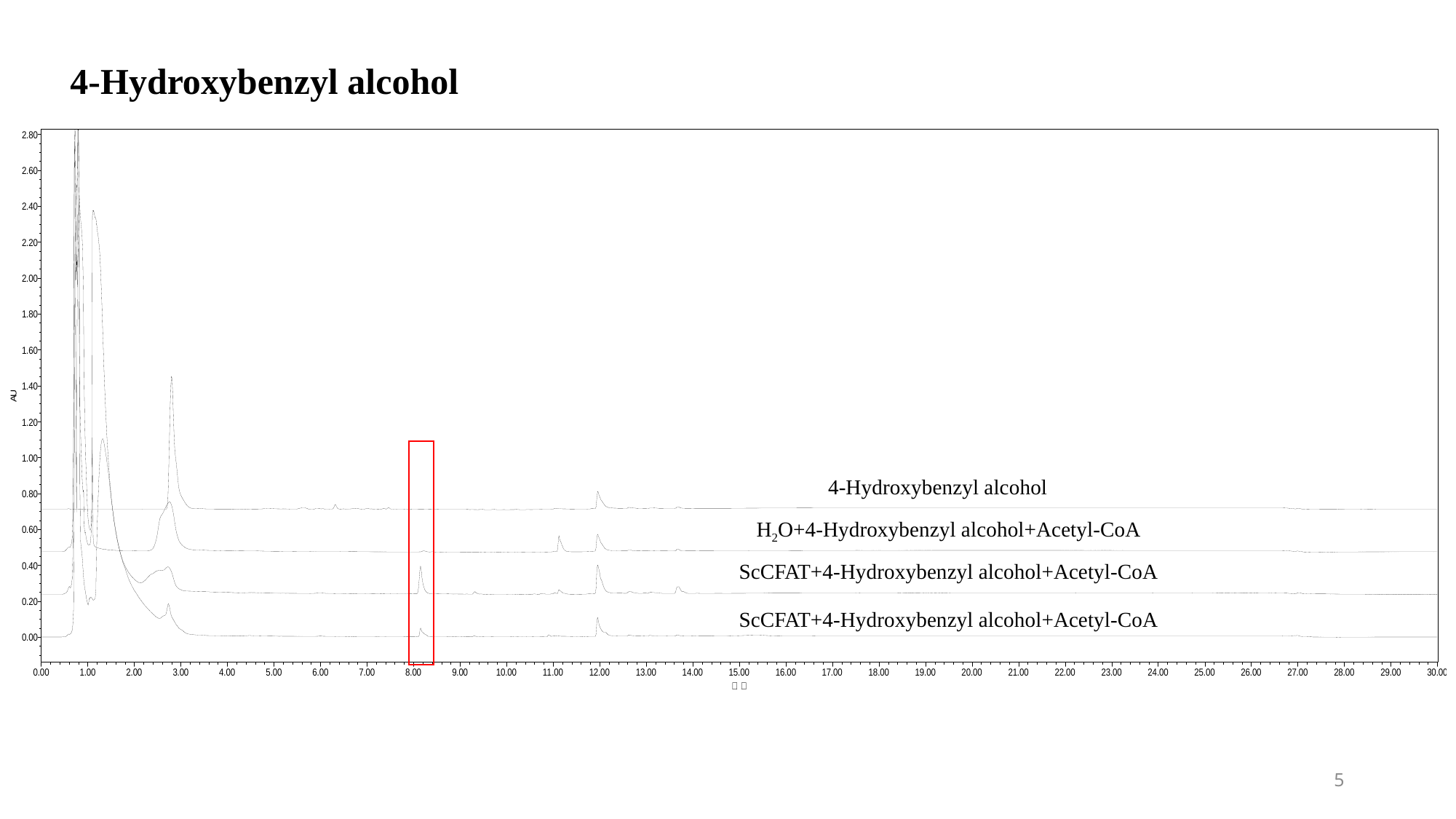

4-Hydroxybenzyl alcohol
4-Hydroxybenzyl alcohol
H2O+4-Hydroxybenzyl alcohol+Acetyl-CoA
ScCFAT+4-Hydroxybenzyl alcohol+Acetyl-CoA
ScCFAT+4-Hydroxybenzyl alcohol+Acetyl-CoA
5

## Slide 6
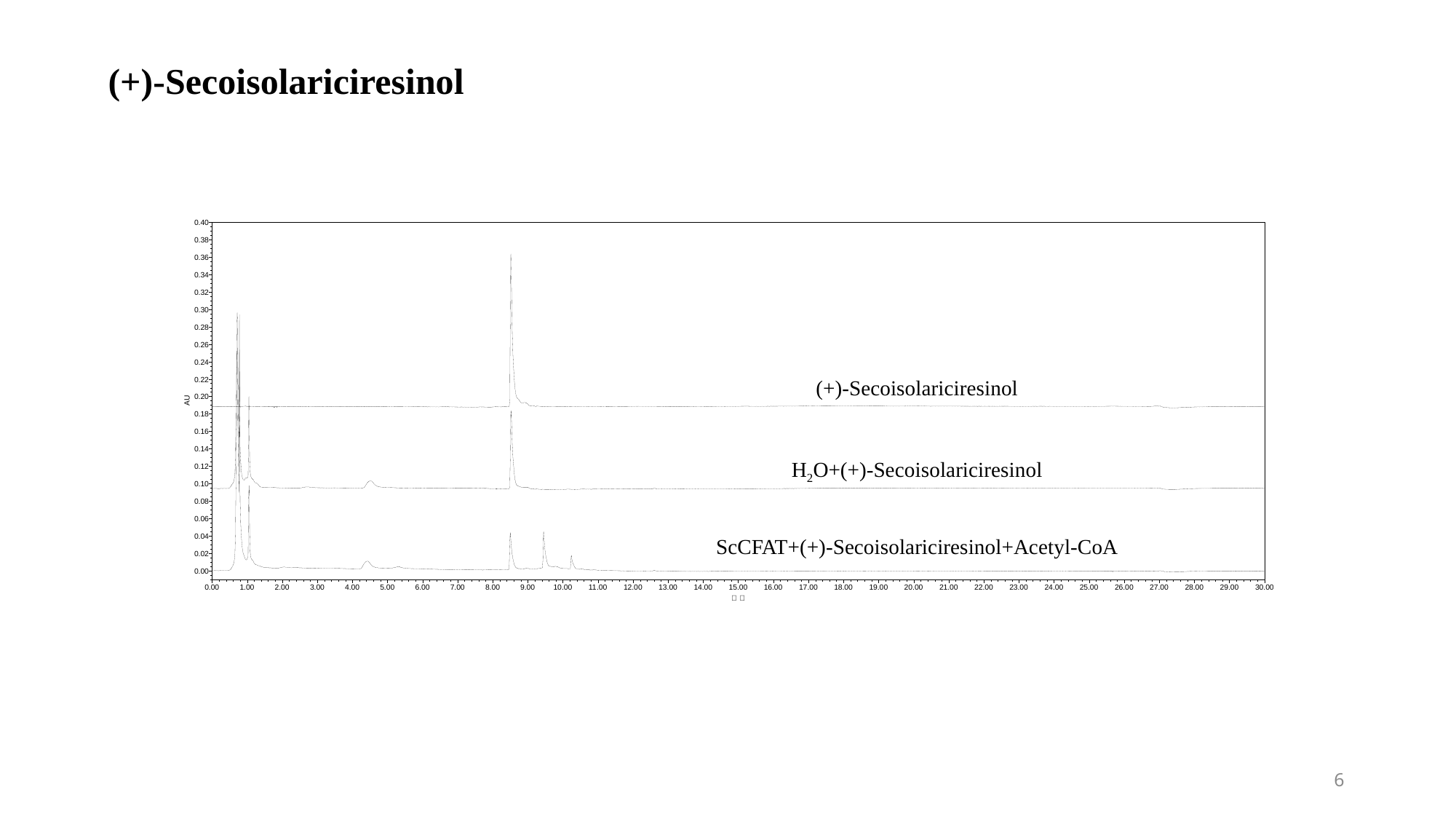

(+)-Secoisolariciresinol
(+)-Secoisolariciresinol
H2O+(+)-Secoisolariciresinol
ScCFAT+(+)-Secoisolariciresinol+Acetyl-CoA
6
