# Supplementary material for: Identification, Molecular Cloning, and Functional Characterization of a Coniferyl Alcohol Acyltransferase Involved in the Biosynthesis of Dibenzocyclooctadiene Lignans in Schisandra chinensis
Source: Front Plant Sci. 2022 Jun 23;13:881342. doi: 10.3389/fpls.2022.881342 (PMC9260284; doi:10.3389/fpls.2022.881342)
Supplement: Supplementary file 8 [file Table_2.DOCX]

**Supplementary Table 2.** Primers used in this study.

| **Primer Name** | **Primer Sequence (5’–3’)** | **Usage** |
| --- | --- | --- |
| ScBAHD1-qF | ACTATGTTACGGCTGAAAACTTATC | RT-qPCR |
| ScBAHD1-qR | GCACTTCTTATCATCGTCGG | RT-qPCR |
| ScGAPDH-qF | CGGTGCTGAGTATGTCGTGGAGT | RT-qPCR |
| ScGAPDH-qR | ACAGTCTTCTGGGTGGCAGTGAT | RT-qPCR |
| ScCFAT-F | ATGGGTCGCGGATCCGAATTCATGGGTATCGCCGGCGGT | Gene cloning |
| ScCFAT-R | TTGTCGACGGAGCTCGAATTCTCAATCTTGATCCAACTTAAGATAGTCA | Gene cloning |
| ScCFAT-LF | ACCATGGTAGATCTGACTAGTATGGGTATCGCCGGCGGT | Subcellular Localization |
| ScCFAT-LR | AAGTTCTTCTCCTTTACTAGTATCTTGATCCAACTTAAGATAGTCAACAC | Subcellular Localization |
